# A novel mechanism of sperm midpiece epididymal maturation and the role of CCDC112 in sperm midpiece formation and establishing an optimal flagella waveform

**DOI:** 10.1186/s12964-025-02320-x

**Published:** 2025-07-01

**Authors:** Maddison L. Graffeo, Joseph Nguyen, Farin Yazdan Parast, Jessica E. M. Dunleavy, Denis Korneev, Hongyi Yang, Hidenobu Okuda, Anne E. O’Connor, Donald F. Conrad, Reza Nosrati, Brendan J. Houston, Moira K. O’Bryan

**Affiliations:** 1 School of Bio Sciences and Bio 21 Molecular Science and Biotechnology Institute, Faculty of Science, The University of Melbourne, Parkville, VIC 3010 Australia; 2 Department of Mechanical and Aerospace Engineering, Monash University, Clayton, VIC 3800 Australia; 3 Department of Biochemistry and Molecular Biology, Monash University, Clayton, VIC 3800 Australia; 4 Reproductive Medicine Center, The First Affiliated Hospital of Zhengzhou University, Zhengzhou, 450018 China; 5 Department of Nephrology and Dialysis, Moriguchi Keijinkai Hospital, Osaka, Japan; 6 Division of Genetics, Oregon National Primate Research Center, Oregon Health & Science University, Beaverton, OR USA

**Keywords:** Spermatogenesis, Mitochondrial sheath, Midpiece, Male fertility, Sperm motility, Epididymal maturation

## Abstract

**Background:**

The mitochondrial sheath is a defining feature of mammalian sperm with proposed functions in structural support and energy production for flagella movement. Recently, coiled coil domain containing (CCDC) protein 112 (CCDC112) was suggested to play a role in the regulation of ciliogenesis. CCDC112 is a poorly characterised protein and there is virtually no knowledge of its in vivo function.

**Methods:**

Here, we define CCDC112 as crucial for male fertility using a *Ccdc112* loss-of-function mouse line. To characterize and analyze male fertility, and to identify a novel process of epididymal midpiece maturation, we utilized a range of assays including fertility testing, scanning electron microscopy, high-resolution sperm motility and power output analysis, in vitro fertilization, intracytoplasmic sperm injection, mitochondria stress test assays and glycolytic flux assays. Localization of CCDC112 in cilia was assessed via the transfection of IMCD-3 cells with a CCDC112-eGFP vector and subsequent immunofluorescent staining.

**Results:**

Results reveal CCDC112 as a requirement for male fertility in the mouse with an essential role in mitochondrial sheath formation. Our data reveal the critical role of CCDC112 in mitochondrial morphogenesis during midpiece formation, with the lack of CCDC112 leading to significantly reduced respiration capacity, irregular flagellar waveforms, diminished progressive motility and ultimately male sterility. In the absence of CCDC112, sperm are unable to traverse the female reproductive tract to the site of fertilization and in vitro have a poor capacity to penetrate the zonae pellucidae of oocytes or fuse with the oocyte. We further unveil a previously unrecognized process of epididymal mitochondrial sheath maturation. We show the sperm midpiece is structurally immature upon exiting the testis and maturation continues during transit from the caput to the cauda epididymis. Finally, we identify CCDC112 as a component of the distal appendages of the mother centriole in IMCD-3 cells suggestive of a facilitative role for CCDC112 in protein entry into the ciliary compartment within germ cells.

**Conclusion:**

Collectively, we establish CCDC112 as a key regulator of sperm midpiece assembly and function while further expanding our understanding on functional sperm production, energy generation and flagella kinematics.

**Supplementary Information:**

The online version contains supplementary material available at 10.1186/s12964-025-02320-x.

## Background

Mammalian sperm are streamlined, highly polarized cells, optimized for traversing the female reproductive tract, locating and fertilizing an ovum. The sperm tail is essential to this process [1]. The sperm tail is a modified flagellum composed of a central microtubule-based axoneme, which functions as the ‘drive shaft’, surrounded by three region-specific accessory structures: the outer dense fibers (mid- and principal pieces), the mitochondrial sheath (midpiece) and the fibrous sheath (principal piece). The processes underpinning sperm assembly remain poorly defined at a molecular level but involve a series of complex microtubule-based protein and organelle transport systems [2]. The mitochondrial sheath, a core component of the midpiece, is hypothesized as essential for structural support [3, 4] and as a source of ATP, via oxidative phosphorylation, to fuel axoneme movement and thus sperm motility [5, 6]. The precise role of mitochondria generated ATP in sperm function and the extent to which it complements glycolytic activity in the principal piece, however, remains controversial [5–10]. Such controversy arises in part due to differences in assay conditions between studies [11] and likely genuine differences in sperm metabolism between species [5, 9, 12–18].

In addition to mitochondrial ATP generation in the midpiece, sperm also produce ATP by glycolysis in the principal piece [19–21]. In somatic cells, glycolysis and oxidative phosphorylation are usually tightly coupled to maximize ATP production [22]. Conversely, in sperm, the majority of glycolytic enzymes are anchored to fibrous sheath in the principal piece, and to a lesser extent in the sperm head and midpiece [19–21, 23, 24]whereas oxidative phosphorylation occurs solely in the midpiece. As such, dogma suggesting they function separately. Recent data, however, challenges this notion [7, 8].

The formation of the mitochondrial sheath at an organelle level has been described, initiating via the recruitment, migration, and successive alignment of spherical mitochondria from the cytoplasmic lobes of elongated spermatids toward the axoneme [25, 26]. Mitochondria then attach to the outer dense fibers surrounding the axoneme along the portion that will ultimately become the midpiece. They then undergo dramatic morphological modifications, transforming from individual spherical organelles into a double helix of rod-shaped mitochondria that abut end to end in a compacted sheath [25, 26]. How the mitochondrial sheath is assembled at a molecular level and its importance to male fertility, however, remains poorly defined. What is clear is that abnormalities in mitochondrial sheath structure are associated with mammalian male infertility including in humans [27–29] and mice [27, 30–34]. Equally, studies in humans and mice have shown that sperm midpiece length is positively associated with sperm swimming speed [35, 36]. Mitochondrial volume has also been correlated with swimming speed, flagellar beat frequency and ATP content [35, 37].

The coiled coil domain containing (CCDC) protein superfamily is a large group of diverse proteins characterised by their coiled coil domains, which are comprised of two or more entwined alpha helices [38]. They function by facilitating protein-protein interactions, potentially acting as molecular spacers by scaffolding macromolecular complexes or separating functional domains in a wide range of biological contexts including during cilia and flagella biogenesis [39–44]the centrosome [39, 45, 46] and fertilization [44]. One such member, CCDC112, has recently been proposed as a ciliation factor in RPE-1 somatic cells, colocalizing with PCM1 in the pericentriolar material [47, 48]. Depletion of CCDC112 in RPE-1 cells resulted in perturbed centriolar satellite fluorescence signal intensity [47]. Single cell sequencing data revealed CCDC112 is highly expressed in pachytene and diplotene spermatocytes in human testes [49].

Herein, we test the function of CCDC112 in vivo, demonstrating an essential role for CCDC112 in male fertility, specifically in mitochondrial morphogenesis and remodeling during mitochondrial sheath formation. Loss of CCDC112 function results in male sterility due to a highly abnormal sperm mitochondrial sheath structure, reduced ATP production, and an irregular flagella waveform with insufficient mechanical power to drive progressive motility and fertilization. We further identify CCDC112 as a distal appendage component of the mother centriole, thus identifying a potential mechanism for the development of a sub-population of short sperm in the absence of CCDC112. Finally, we reveal a previously unrecognized form of epididymal sperm maturation, epididymal mitochondrial sheath maturation. Collectively, these data identify CCDC112 as a critical regulator of sperm midpiece assembly and function and uncover a novel regulator of mitochondria morphology.

## Methods

### Loss-of-function mouse model production

All animal procedures were conducted in accordance with the animal ethics guidelines generated by the Australian National Health and Medical Research Council (NHMRC). Experimental procedures were approved by The University of Melbourne Animal Ethics Committee (application 20640) or Monash University Animal Experimentation Ethics Committee (BSCI/2017/31).

A *Ccdc112* loss-of-function mouse line was generated on the C57BL/6J background through the Monash University Genome Modification Platform (a partner of the Australian Phenomics Network) using CRISPR/Cas9 technology. Guide RNAs targeting regions upstream and downstream of exon 2 (GCAGTGACCGCGCATGCACATGG and TTCCTAATACTGTGAGACTGGGG, respectively) and Cas9 machinery were injected into fertilized mouse eggs (zygotes) to target the removal of exon 2 of *Ccdc112* of the principal (longest) transcript (*ENSMUST00000072835.7* [2801 bp]). The 122 bp deletion was confirmed via Sanger sequencing. This modification led to a premature stop codon in exon 3 of *Ccdc112* and a non-functional product. Mice heterozygous for the *Ccdc112* deletion were bred to generate loss-of-function individuals (*Ccdc112*^*KO/KO*^) and wildtype controls (*Ccdc112*^*WT/WT*^). Genotyping was performed using the primers (Forward – 5’-GTTCGGAACAGTGTGCGGAG-‘3 and Reverse – 5’-AGCTGGCTTGAGTTCTGCTT-‘3) which amplified a 1050 bp wildtype band and 680 bp loss-of-function band. The effect of exon deletion on *Ccdc112* mRNA expression was assessed using quantitative polymerase chain reaction (qPCR) on testis cDNA using primers spanning exons 4/5 to 6 of transcript *Ccdc112-201* (principal transcript; *ENSMUST00000072835.7* [2801 bp]) and *Ccdc112-203* (ENSMUST00000236934.2 [643 bp]) (Forward – 5’- GCTGTAGTGCTTCAGGTGG -‘3 and Reverse – 5’- AGTCCCTCTTTGGTTGTAG -‘3) and exons 7 to 9 of *Ccdc112-205* (ENSMUST00000238189.2 [761 bp]) (Forward – 5’- CGGATGAGCTTGCCAGGTTTC -‘3 and Reverse – 5’- GTAGGTTTGTAAAGCCTAGAAGGATCTC -‘3). qPCRs were performed using SYBR Select Master Mix (Applied Biosystems). Each reaction was performed in triplicate for wildtype and quadruplicate for *Ccdc112*^*KO/KO*^ using the Agilent Mx3000P qPCR system or an Applied Biosystems QuantStudio 3 Real-Time PCR System with the parameters of 50°C for 2 minutes, 95°C for 10 minutes followed by 40 cycles of 95°C for 15 seconds and 60°C for 1 minute. *Ppia* was amplified at the same time as a housekeeping control (using 5’-CGTCTCCTTCGAGCTGTTT-’3 forward and 5’-CCCTGGCACATGAATCCT-’3 reverse primers), and all results were normalized to *Ppia* expression. Differential expression was analyzed using the 2ΔΔCT method [50].

### Analysis of *Ccdc112* expression

To investigate the expression of *Ccdc112* in human tissue, we obtained single cell RNA sequencing data from [51]. To assess expression in human and wildtype mouse testis cells we obtained single cell RNA sequencing data from ^52^ to ^53^, respectively. Usage of these data sets was done in accordance to the Creative Commons Attribution 4.0 International License (http://creativecommons.org/licenses/by/4.0/).

### Fertility characterization

Male fertility in *Ccdc112* loss-of-function male mice, compared to wildtype colony mates, was assessed as per the framework described in [54]. Briefly, male mice at 10–12 weeks of age were mated with two wildtype females (40–80 days of age). Successful mating was confirmed by the presence of copulatory plugs, and litter size was recorded as the number of pups born per plug. Males were subsequently culled and weighed. For histological examination, testes and epididymides were dissected, weighed, and fixed in Bouin’s fixative for 5 h, processed into paraffin blocks, and cut into 5 μm sections using standard methods. For sperm motility assessment, cauda epididymal sperm were backflushed into MT6 medium at 37 ^o^C, allowed to disperse for 15 min, then examined via computer assisted semen analysis (CASA) (*n* ≥ 1000 cells/animal; *n* ≥ 3 animals/genotype) [55, 56].

Periodic acid-Schiff’s reagent and hematoxylin staining was undertaken and used to visualize testis histology (*n* ≥ 5/genotype), while epididymis histology and sperm morphology were investigated using hematoxylin and eosin staining (*n* ≥ 3/genotype). Epididymal sperm morphology was assessed and broadly categorized as “normal” as determined by the most common sperm phenotype observed in wildtype mice or “abnormal”, then further categorized into cellular defects, including “abnormal sperm head morphology”, “abnormal sperm midpiece structure”, “short tail” and/or “coiled tail” (*n* ≥ 100 sperm/animal; *n* = 3 animals/genotype). Mitochondrial sheath, midpiece and tail lengths were measured using Fiji (version 2.9.0/1.53t) (*n* = 3–5 animals/genotype for midpiece, mitochondrial sheath and tail length; *n* ≥ 100 sperm/animal for tail length, *n* ≥ 20 sperm/animal for mitochondrial sheath length and *n* ≥ 30 sperm/animal for midpiece length). Mitochondrial sheath and tail length were determined based on hematoxylin and eosin stained cauda epididymal sperm and measured from the base of the sperm head to end of discernable sheath and to the tip of the tail, respectively. Midpiece length was determined based on cauda epididymal sperm stained for annulus marker, SEPT4 and measured from the base of the sperm head to the annulus.

Testis daily sperm production (DSP) and epididymal sperm content (ESC) were determined on snap frozen tissues using a modified version of the Triton X-100 nuclear solubilization method as previously described in [57] on mice aged 10–12 weeks of age (*n* ≥ 3/genotype). Briefly, to lyse all uncondensed cells and remove residual sperm tails, snap frozen testes or epididymides were thawed in 1 ml DSP buffer (0.9% NaCl, 0.01% NaN_3_, 0.05% Triton X-100). Samples were then homogenized via sonication using a 125 W probe sonicator (Q125 Sonicator, Qsonica) at 30% amplitude for three 10 s pulse cycles at 10 s intervals. The number of condensed (detergent and sonication resistant) elongated spermatids per testis/sperm per epididymis was counted using a hemocytometer. For DSP rates, the number of elongated spermatids was multiplied by 4.84; the number days elongated spermatids are present in the mouse testis [58]. ESC is presented as an absolute number.

Quantification of the percentage of stage IX tubules with retained spermatids, compared to wildtype, was conducted on at least 10 PAS-stained stage IX tubules per animal (*n* = 3 animals/genotype). Quantification of round spermatid number per stage VIII tubule was conducted on 10 tubule cross-sections wherein the cross-section was round as an indication of dissection perpendicular to the length of the tubule (*n* = 5 animals/genotype). Germ cell apoptosis was examined via immunostaining for cleaved caspase 3 and 7 as outlined in [57]. The number of caspase positive cells was counted in a minimum of 100 randomly selected tubules per mouse (*n* = 5 animals/genotype).

To assess the ability of sperm to traverse the female reproductive tract and reach the oviduct, wildtype and *Ccdc112*^*KO/KO*^ male mice were plug mated overnight with wildtype females. Before 9 am plugs were checked and upon detection of a positive plug females were culled at 10 am (an estimated 10 h post-mating). Oviducts were dissected to separate the isthmuses and ampullae. Each pair of oviduct regions was then minced and dried onto a glass slide after removing large chunks of tissue, then fixed with 4% paraformaldehyde in PBS (10 mM Na_3_PO_4_, 2.68 mM KCl, 140 mM NaCl) for 10 min before being washed in PBS. Sections were stained with hematoxylin and eosin and the number of sperm per region counted (*n* ≥ 3/genotype).

### Electron microscopy

To investigate caput and cauda sperm mitochondrial sheath structure via scanning electron microscopy, sperm were isolated from the caput epididymis by careful dissection and nicking of the cauda epididymis with a sharp scalpel blade in warm PBS in a Petri dish at 37 ^o^C for 30 min. For the cauda epididymis, sperm were collected using the backflushing method [59]then incubated in 100 µl of 1 × PBS (10 mM Na_3_PO_4_, 2.68 mM KCl, 140 mM NaCl) for 30 min to remove of the plasma membrane [60]. Scanning electron microscopy was undertaken as described in [60]. Plasma membrane removal was highly effective, with membrane intact sperm rarely seen for both genotypes and epididymal regions. To investigate cauda epididymis sperm ultrastructure via transmission election microscopy, cauda epididymal sperm were backflushed into MT6 medium before being processed as previously described [57]. Images were captured on a Talos L120C for transmission electron microscopy or a FEI Teneo VolumeScope for scanning electron microscopy at the Ian Holmes Imaging Centre (The University of Melbourne, Australia) or a Jeol 1400 Plus electron microscope for transmission electron microscopy at the Vera and Clive Ramaciotti Centre for Electron Microscopy (Monash University, Australia).

### Scoring of mitochondrial sheath normality

After noticing abnormalities in mitochondrial sheath structure in sperm from *Ccdc112*^*KO/KO*^ males, the degree of sheath abnormality was quantified on scanning electron microscopy images (*n* ≥ 5 mice/genotype). For each biological replicate, a minimum of 10 sperm from each region of the epididymis were assessed and scored from 1 [highly abnormal] to 7 [normal]. We defined these categories as: 1 – mitochondria were completely absent or the mitochondrial sheath was stratified (highly elongated mitochondria morphed together); 2 – mitochondria were frequently immature, morphed together, incorrectly oriented or sections of the sheath lacked mitochondria; 3 – mitochondria were often immature (round/crescent), morphed together and/or incorrectly oriented; 4 – many mitochondria had abnormal orientations and size, including mitochondria morphed together (beginning and end of mitochondria was not discernible); 5 – mitochondria were loosely coiled with some incorrectly oriented (more horizontal, or diagonal orientation – i.e., less perpendicular to the axoneme) and heterogeneously sized mitochondria; 6 – mitochondria were less tightly coiled and/or a small section of the sheath bearing mitochondria had misaligned ends; 7 – no defects, with mitochondria in the sheath being tightly packed, regularly coiled, homogenously sized and correctly orientated (i.e., almost perpendicular to the axoneme). Using a strict rating system, a score of 1–6 was considered abnormal i.e. displayed disordered mitochondrial packing. To ensure the rating system used was unbiased and reproducible, two biological replicates for each genotype were blindly assessed and rated by a second researcher and found to match the original score. To further define the disordered mitochondrial sheath packing of caput sperm, defects were separately classified to define the incidence of immature mitochondria (spherical and/or crescent shaped), exposed outer dense fibers and/or no mitochondria.

### High resolution sperm motility analysis

To assess the kinematics of sperm flagellar movement, beating patterns of head-tethered motile cauda epididymal sperm were recorded and analyzed at high resolution. Video capture and analysis was undertaken as outlined previously [61]. Briefly, sperm were backflushed from the cauda epididymis into modified TYH medium (10 mM HEPES, 5.6 mM glucose, 1 mM MgSO_4_, 1.2mM KH_2_PO_4_, 2 mM CaCl_2_, 4.8 mM KCl, 135 mM NaCl, 0.5 mM sodium pyruvate, 10 mM sodium L-lactate), supplemented with 0.3 mg/ml of bovine serum albumin at 37 ^o^C. After 15 min, sperm were diluted 1 in 10 in TYH medium. A custom-made imaging chamber was made by affixing two strips of clear adhesive tape 15 mm apart on a glass microscope slide. 10 µl of the diluted sperm suspension was added in between the tape and covered with a glass coverslip (Menzel Gläser). Sperm motility patterns were then imaged using an Olympus AX-70 upright microscope outfitted with a U-DFA 18 mm internal diameter dark-field annulus and a 20 × 0.7 NA objective (UPlanAPO, Olympus, Japan). Images were captured via a Zyla 5.5 CL10 sCMOS camera (Andor Technology, Northern Ireland) at 250 frames per second using the Fiji image-processing package with the Micro-Manger Studio plugin (version 1.4.23). Ten videos per animal (*n* = 5 animals/genotype) capturing sperm tail movement over 1000 frames were taken. Images were then adjusted and processed in Fiji (version 2.9.0/1.53t) before being analyzed in MATLAB (version R2021b) using a modified version of the MATLAB codes used in [61] and publicly available from the Monash University Research Repository (DOI: 10.26180/14045816). Briefly, analysis of each image was conducted by extracting the sperm flagella centerline and reconstructing its waveform using an automated image analysis algorithm as previously described in [61]. The curvature kymograph was plotted by calculating the tangent angle of the flagella waveform. The beating frequency of the sperm flagella was calculated by determining the average frequency of curvature turning points at 50% and 75% of the flagella length. The mean sperm flagella amplitude of the midpiece and principal piece was calculated by using set points corresponding to the sperm head (6.3 μm), mid (22.4 μm) and principal (80 μm) piece lengths [35] and determining the average amplitude along each tail section. Analysis of the flagella power (motor input power and dissipated powers) for each image, were calculated as previously described in [61]. Briefly, motor input was defined as power expended from the dynein motors towards the sperm flagellum [62]. Motor dissipation was defined as energy dissipated by the dynein motors due to work done of them from other flagella structures, internal dissipation was defined as energy dissipation due to friction between flagellum cross sectional planes and hydrodynamic dissipation was defined as energy dissipation into the fluid surrounding the flagellum.

Flagella waveform graphs were categorized into 4 main groups based on their midpiece amplitude. Midpieces with an amplitude ranging from 27 to 39 μm were considered highly flexible, 16–27 μm were relatively flexible, 6–15 μm were moderately stiff and 0.6–6 μm were highly stiff.

### Assisted fertility methods

To assess the ability of sperm to interact with and fertilize oocytes, in vitro fertilization using zonae pellucidae intact and zonae pellucidae stripped oocytes was undertaken. To induce superovulation, 3–5-week-old C57BL6/CBA (F1) female mice were given 100 µl (5 IU) intraperitoneal injections of equine chronic gonadotrophin (Folligon) followed by 100 µl (5 IU) injections of human chorionic gonadotrophin (hCG – Chorulon) 48 h later. The following day, male mice were culled, and their cauda epididymides dissected into warm mineral oil. Sperm were then backflushed gently into 200 µl pre-equilibrated drops of G-IVF PLUS (Vitrolife, Sweden) under OVOIL in 35 mm Petri dishes and incubated for 1 h at 37 ^o^C in 6% CO_2_ to capacitate. In parallel, 14 h after the second round of injections, female mice were culled, and their ovaries were dissected and placed in 1 m G-IVF PLUS at 37 ^o^C. For zonae pellucidae intact oocytes, cumulus-oocyte complexes were retrieved from the ampulla and washed 3 times in high calcium HTF media (25 mM NaHCO_3_, 5.55 mM glucose, 0.2 mM MgSO_4_.7H_2_O, 0.31 mM KH_2_PO_4_, 5.14 mM CaCl_2_.2H_2_O, 4.69 mM KCl, 101.61 mM NaCl, 2.72 mM sodium pyruvate, 26 mM sodium L-lactate, pH 7.4) supplemented with 4 mg/ml bovine serum albumin (fraction V), 10 µl/ml penicillin-streptomycin and 1 mM reduced glutathione (GSH; G4251, Sigma). For zonae pellucidae stripped oocytes, cumulus-oocyte complexes were retrieved from the ampulla and placed into hyaluronidase (1 mg/ml hyaloyuronidase in G-MOPS; Vitrolife) to remove the cumulus (30 s to 2 min). Cumulus-free oocytes were then washed three times in G-IVF PLUS before being transferred to Acid Tyrodes (T1788, Merck, Germany) for a maximum of 2 min. Oocytes were then immediately washed 3 times in G-IVF PLUS then left for 90 min to recover their surface proteins. Complexes or cumulus-free oocytes were then moved to a fertilization dish of HTF media and GSH covered with OVOIL, to which 5 × 10^5^ sperm were added and incubated at 37 ^o^C in 6% CO_2_, 5% O_2_ for 4 h. Presumptive zygotes were washed in 50 µl drops of pre-equilibrated G1 PLUS medium in 35 mm Petri dishes at 37˚C in 6% CO_2_ for 30 min, then cultured in G1 PLUS overnight. Sixteen-hours post fertilization, the number of 2-cell presumptive embryos was counted, and 2-cell rate determined.

To assess the ability of sperm to fertilize oocytes with all barriers removed, intra-cytoplasmic sperm injection (ICSI) was undertaken. Sperm were collected and capacitation as described above. Induction of superovulation, collection of cumulus-oocyte complexes and stripping of the zonae pellucidae from oocytes from 3 to 5 week old C57BL6/CBA (F1) female mice was conducted as described above. Following incubation in hyaluronidase, cumulus-free oocytes were transferred into G-MOPS (16006, Vitrolife) and sperm were transferred into ICSI media (10111, Vitrolife). To prepare sperm for injection, individual sperm were isolated, struck on the neck with a ICSION injection pipette (I-4.0-30, Australia) to immobilize then struck again to detach the sperm head from the tail. Isolated sperm heads were injected into oocytes using an Olympus IX73 inverted microscope (Japan) at 200x magnification. Here, oocytes and sperm were micromanipulated using the Narishige ON-4 fine manipulator (Japan) and Narishige MM-950 coarse manipulator, oocytes were stabilized using an ICSION Holding pipette (H-25/120 − 30) paired with the Eppendorf Celltram 4r Air microinjector (Germany), and sperm were injected using an injection pipette paired with the Eppendorf Celltram 4r Oil microinjector. Oocytes were then washed and incubated overnight as above. Sixteen-hours post-injection, the number of 2-cell presumptive embryos was counted, and 2-cell rate determined.

### Immunochemistry

To assess protein localization in the paraffin embedded testis sections, sections were processed and stained as previously described using diaminobenzidine (DAB) as the chromogen [63].

To assess protein localization in cultured cells, cells were fixed in cold 100% methanol, before non-specific antibody binding was blocked with CAS-block, incubated with primary and secondary antibodies, counterstained with DAPI and mounted, as above.

As above, to assess midpiece length in mature sperm, backflushed cauda epididymal sperm were settled in PBS onto slides overnight. Sperm were fixed in 1:1 methanol to acetone for 2 min, washed in PBS, permeabilized in 0.2% Trition-X for 20 min. Following, sperm were washed in PBS, before being blocked with CAS-block, incubated with primary and secondary antibodies, counterstained with DAPI and mounted, as above.

To examine oocytes 16 h post-fertilization, cells were fixed in 4% PFA for 30 min, washed in PBS, permeabilized in 0.2% Trition-X for 1 h. Following, cells were washed in PBS, stained with DAPI for 20 min, before being washed in PBS and mounted in µ-slide 8 well glass bottom plate (80827, Ibidi Gmbh, Germany).

Primary antibodies used included those against α-tubulin (T5168, Sigma; 0.1 µg/ml), CEP170 (ab84545, Abcam, USA; 1 in 500), cleaved-Caspase 3 (9664, Cell Signaling; 0.5 µg/ml), cleaved-Caspase 7 (9491, Cell Signaling; 0.23 µg/ml), GFP (11814460001, Roche, Switzerland; 0.4 µg/ml), ODF2 (HPA001874, Sigma; 0.28 µg/ml), SEPT4 (166788, Abcam; 2.5 µg/ml) and γ-tubulin (ab11317, Abcam; 11.7 µg/ml). Trialed commercial CCDC112 primary antibodies included HPA045120 (Sigma), HPA050621 (Sigma) and ab237742 (Abcam). Secondary antibodies were used diluted 1 in 500 and included Alexa Fluor 488 donkey anti-mouse (ab150105, Abcam) and Alexa Fluor 555 donkey anti-rabbit (ab150074, Abcam).

Brightfield images were collected on the Olympus BX53, Olympus DP80 camera with the Olympus Cell Sens Dimension software (v3.1.1). Immunofluorescent images were captured using either a Leica TCS SP8 confocal microscope (Leica Microsystems) or the Elyra LSMS880 (Zeiss, Germany) in the Biological Optical Microscopy Platform facility (University of Melbourne) or an Abberior STED-Super-Resolution Microscope (Abberior, Germany) at the Monash Micro Imaging Facility (Monash University). All immunofluorescent images were taken using the 63x/1.40 HC PL APO CS2 oil immersion objective besides oocyte images which were taken using the 20x/0.8 Plan-Apochromat air objective. Images were adjusted uniformly across the image and between groups.

### Mitochondrial membrane potential

Mitochondrial membrane potential was analyzed using mitochondrial fluorescent probe JC-1 (T3168, Invitrogen) using a modified version of the analysis previous described in [64]. Briefly, sperm were backflushed from the cauda epididymis into MT6 medium (excluding phenol red) at 37 ^o^C. Sperm were then incubated with 1 µM JC-1 in MT6 (T3168, Invitrogen) for 20 min at 37 ^o^C in the dark. Simultaneously, LIVE/DEAD staining was performed (L7011, Invitrogen) by incubating cells with SYBR14 (1 µl/ml) and 5 µM propidium iodide (PI) for 10 min at 37 ^o^C in the dark. To do so, PI and SYBR 14 dye was added halfway through JC-1 incubation. Following incubation, cells were centrifuged for 1 min at 300 × g and then gently resuspended in fresh MT6 medium before being imaged. To prevent sperm swimming out of frame during imaging, custom-made laminin coated observation chambers were created. To do this, two strips of clear tape were affixed at either end of a glass slide, before 0.2 mg/ml laminin (Thermo Fisher Scientific, USA) in TBS was added to slides and allowed to coat for 1 h at 37 ^o^C in a humified chamber. After incubation, slides were washed with water and kept warm until used. Per slide, 100–150 µl of the sperm suspension was added in between the tape and covered with a glass coverslip (Menzel Gläser, thickness 1). Sperm were then imaged using filters green (FITC), red (CY3) and far-red (PI) on an Olympus BX53, Olympus DP80 camera with the Olympus Cell Sens Dimension software (v3.1.1). Mitochondrial membrane potential of sperm was then quantified on live cells (PI/far-red negative) by measuring the fluorescence intensity of the red channel (high membrane potential) relative to the green channel (low membrane potential) using Imaris (10.0.1), *n* ≥ 20 sperm/animal; *n* = 3 animals/genotype).

### Sperm mitochondrial respiration and glycolytic flux analysis

To assess sperm metabolism, Seahorse real-time metabolic analysis was undertaken. First, the assay cartridge was prepared. The day prior to the assay, the Seahorse sensor cartridge was hydrated in sterile water in a non-CO_2_ 37 °C incubator overnight. Following hydration, the sensor cartridge was then calibrated via incubation in pre-warmed Seahorse XF Calibrant (incubated overnight in a non-CO_2_ 37 °C incubator) for 45–60 min prior to loading the injection ports of the sensor cartridge. After incubation, the cartridge was removed from the incubator and each port was loaded as follows: For the mitochondrial stress test assay – 1st injection port – 20mM Oligomycin A (75351, Sigma); 2nd injection – 20µM FCCP (C2920, Sigma); 3rd injection port – 10mM Rotenone (R8875, Sigma) and 10mM Antimycin A (A8674, Sigma) and 4th injection – a modified TYH media ((2 mM HEPES, 5.6 mM glucose, 1.2 mM MgSO_4_, 1.2 mM KH_2_PO_4_, 1.7 mM CaCl_2_, 4.7 mM KCl, 138 mM NaCl), supplemented with 3 mg/ml of bovine serum albumin). For the glycolytic stress test assay – 1st injection port – 100mM D-Glucose (A783, Univar, USA); 2nd injection port – 20mM Oligomycin A; 3rd injection port – 500mM 2-Deoxy-D-Glucose (L07338.06, Thermo Fisher Scientific) and 4th injection port – a modified TYH media ((1 mM HEPES, 1.2 mM MgSO_4_, 1.2 mM KH_2_PO_4_, 1.7 mM CaCl_2_, 5.4 mM KCl, 144 mM NaCl), supplemented with 3 mg/ml of bovine serum albumin). These injections are then diluted 10 fold to leave the final concentrations for the mitochondrial stress test assay at 2mM oligomycin A, 2mM FCCP, 1mM Rotenone and 1mM Antimycin A and for the glycolytic stress test assay at 10mM D-Glucose, 2 μm oligomycin A and 50mM 2-Deoxy-D-Glucose. The cartridge was then transferred to the XFe96/XF96 instrument and calibration via the Wave software (version 2.6.3) began. In parallel, to ensure sperm heads adhered to the Seahorse plate, each plate well was incubated with 30 µl of mouse laminin diluted with sterile filtered TBS (0.2 mg/ml; 23017015, Thermo Fischer Scientific) at 4^o^C overnight. The following day, the laminin coated Seahorse plate was incubated in a non-CO_2_ 37 °C incubator for 1.5 h. The majority of the laminin was then removed by pipetting, leaving a thin coat, and the plate rinsed with MilliQ water just prior to the addition of sperm. For the assay, cauda epididymal sperm were backflushed into 1 ml of a modified TYH medium at 37 ^o^C. After 15 min, sperm in solution were transferred to a new pre-warmed microfuge tube. 1.2 million sperm per well were then added to the Seahorse plate. The plate was then spun with no braking in a prewarmed 37 ^o^C centrifuge at 300 g for 5 min. Cells were than rested in a non-CO_2_ 37 °C incubator for 15 min prior to starting the assay. The Seahorse plate was then loaded into the XFe96/XF96 instrument following Seahorse sensor cartridge calibration and analysis was conducted using the Wave software. At the end of the experiment the number of sperm per well was then re-counted using a hemocytometer to avoid bias from differential adhesion. Results were normalized accordingly.

### Cell culture and transfection

To examine the localization of CCDC112 in cilia, IMCD-3 cells (ATCC) were transfected with a CCDC112-EGFP vector. The expression vector was constructed by inserting a copy of the *Ccdc112* mouse cDNA (Forward – 5’-GTCTCGACGAGGAAACATGG-‘3 and Reverse – 5’-TAGGAACAGAACTGACAATGCTC-‘3), amplified from wildtype C57BL/6J testis cDNA by PCR reaction, into a pEGFP-C1 expression vector. IMCD-3 cells were cultured in DMEM/F12 medium supplemented with 10% FBS and 1% penicillin and streptomycin. The CCDC112-EGFP vector was then transfected into IMCD-3 cells using Lipofectamine 2000 Reagent (Thermo Fisher Scientific). Briefly, cells were seeded and incubated as per the manufacturer’s instructions. Ciliogenesis was then induced by culturing IMCD-3 cells on coverslips in OptiMEM serum free medium (Thermo Fisher Scientific) for 48 h as previously reported [65, 66]. Cells were then fixed, stained, and imaged as detailed above.

### Statistics

Statistical analyses were conducted in GraphPad Prism 10 (version 10.0.3) with the statistical difference between genotypes determined using an unpaired student’s T-test on averages of technical replicate data per animal. The statistical difference between more than two groups was determined using a two-way ANOVA and Tukey post-hoc analysis, respectively. Significance was defined as *p* < 0.05. Specifically, statistical differences for breeding data, weights, DSP, ESC, the percentage of stage IX tubules with retained spermatids, round spermatids per tubule numbers, oviduct counts, CASA and high-resolution sperm motility analysis, high-resolution sperm power outputs, sperm midpiece, mitochondrial sheath and tail lengths, sperm morphology (brightfield), caput midpiece comparison, in vitro fertilization, ICSI, mitochondria Seahorse stress test assay, glycolytic flux Seahorse assay and mitochondrial membrane potential, an unpaired student’s T-test was used. For the remaining mitochondrial sheath abnormality comparisons (normal mitochondrial sheath data, rating of midpiece abnormality severity and relative sperm count) a two-way ANOVA was used. Statistical analysis of germ cell apoptosis data was conducted using generalized linear mixed models with zero-inflated negative binomial distribution as determined by the Akaike information criteria estimates [67]. Analysis was undertaken in R version 4.1.10 (R Core Team 2021) and R Studio version 1.4.1717 (RStudio Team 2020) using the lme4 [68] and glmmTMB [69] packages.

## Results

### CCDC112 is testis-enriched

*CCDC112* is a highly conserved protein between human (ENSG00000164221) and mouse (ENSMUSG00000071855) with 85% protein sequence alignment. A previously published analysis of *CCDC112* expression in human organs revealed it was highly enriched within testes compared to other tissues (*p* < 0.0001; Fig. S1A) [51]. Further, single cell RNA sequencing data of germ cells revealed that in humans *CCDC112* is most highly expressed in late primary spermatocytes and to a lesser extent in spermatogonia (Fig. S1B) [52]. A similar expression pattern was observed in mouse male germ cells (Fig. S1C) [53]. Unfortunately, and despite assessing four CCDC112 antibodies, including one produced in-house, all antibodies tested in this study bound non-specifically to other proteins in immunochemistry and western blotting protocols. As such, CCDC112 localization could not be defined.

### CCDC112 is essential for male fertility

To explore the role of CCDC112 in male fertility, we produced a *Ccdc112* loss-of-function mouse model (*Ccdc112*^*KO/KO*^) whereby we removed exon 2 from the principal *Ccdc112* mouse transcript (*ENSMUST00000072835.7)*, which introduced a premature stop codon in exon 3 (Fig. S1F, G). As outlined in Ensembl, *Ccdc112* has three transcripts (Fig. S1G). Deletion of exon 2 will target transcripts *Ccdc112-201* and *Ccdc112-203*, wherein *Ccdc112-201* is predicted to be the primary transcript, and will lead to a premature stop codon in exon 3. *Ccdc112-205* contains exons 6–7 and 9–10. qPCR targeting transcripts *Ccdc112-201* (principal) and *Ccdc112-203* measured a 99.8% reduction in *Ccdc112* mRNA expression in mutant testes compared to wildtype (Fig. S1E). qPCR targeting transcripts *Ccdc112-205* and *Ccdc112-201* (noting overlapping exon content) measured a 214% increase in *Ccdc112* mRNA expression in mutant testes compared to wildtype littermates. Collectively these data suggest the up-regulation in transcript *Ccdc112-205*, in the absence of *Ccdc112-201*. An examination of the sequence within *Ccdc112-205* indicate that it is unlikely to produce a functional protein as the start and stop codons are located only 42 base pairs (14 amino acids) apart within the first exon (exon 6; Fig. S1H).

**Fig. 1:**
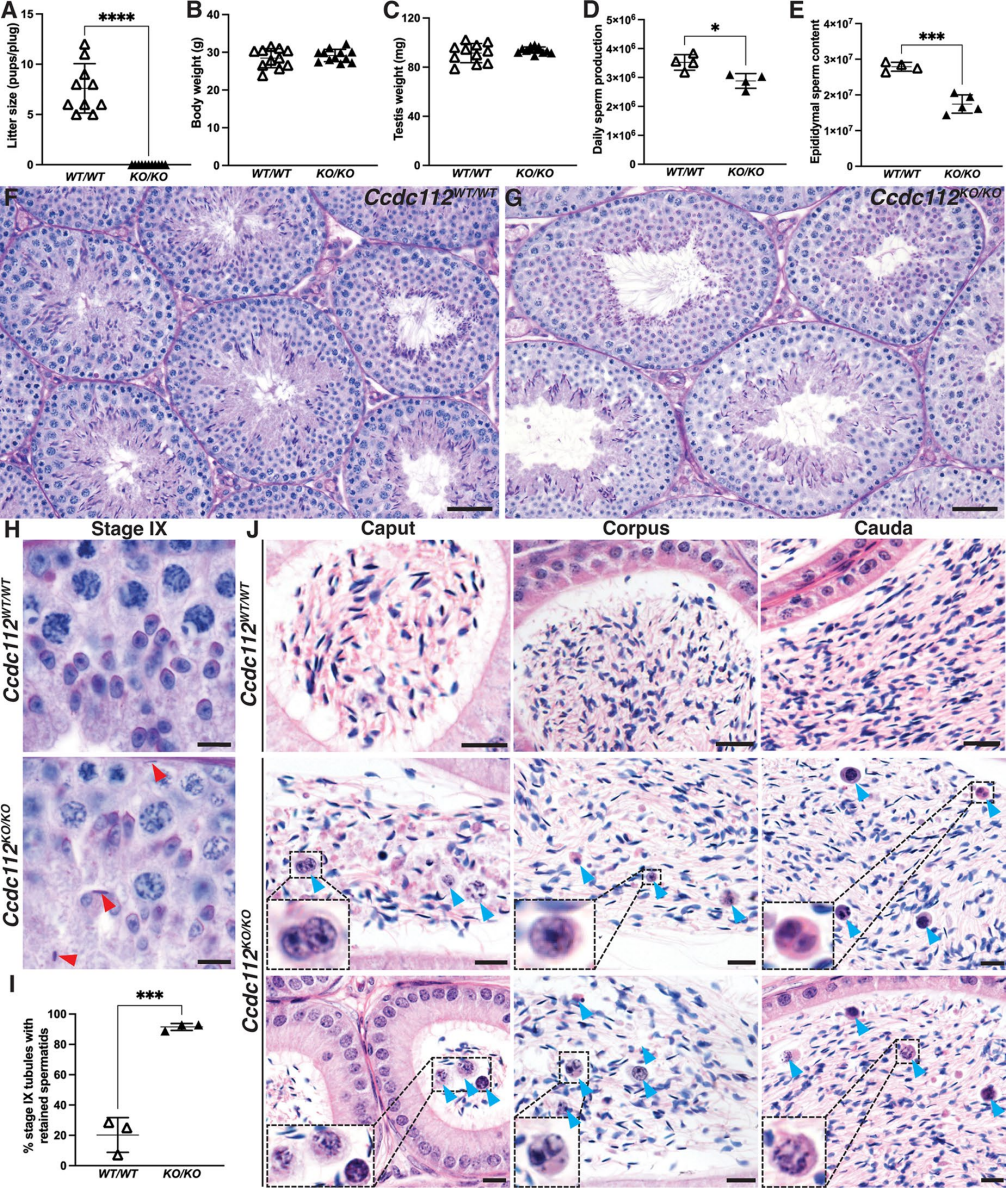
CCDC112 is required for male fertility Litter size (**A**), body weight (**B**), testis weight (**C**), testis daily sperm production (DSP) (**D**) and total epididymal sperm count (ESC) (**E**) in *Ccdc112*^*WT/WT*^ mice and *Ccdc112*^*KO/KO*^ mice (*n* ≥ 4/genotype). Periodic acid-Schiff stained testis sections (**F**-**H**) and hematoxylin and eosin-stained epididymis sections (**J**) from *Ccdc112*^*WT/WT*^ and *Ccdc112*^*KO/KO*^ mice. Red and blue arrowheads indicate retained spermatids in (**H**) and prematurely sloughed germ cells in (**J**), respectively. (**I**) Quantification of the percentage of stage IX tubules with retained spermatids in *Ccdc112*^*WT/WT*^ and *Ccdc112*^*KO/KO*^ mice (*n* ≥ 9/tubules and *n* = 3/genotype). Lines denote mean ± SD in A-E, I; * *P* < 0.05, *** *P* < 0.001, **** *P* < 0.0001. Scale bars in F-G = 50 μm, H = 10 μm, J = 20 μm

*Ccdc112*^*KO/KO*^ mice appeared outwardly normal with no overt behavioral abnormalities and normal mating frequency. They were, however, male sterile (0 pups per copulatory plug versus 7.5 in *Ccdc112*^*WT/WT*^, *p* < 0.0001; Fig. 1A). Body weights (Fig. 1B) were comparable between *Ccdc112*^*WT/WT*^ and *Ccdc112*^*KO/KO*^ mice. While testis weights were unchanged between genotypes (Fig. 1C), testis daily sperm production (DSP) and epididymal sperm content (ESC), were reduced by 13% (*p* < 0.05) and 38% (*p* < 0.001), respectively, in *Ccdc112*^*KO/KO*^ males compared to wildtype (Fig. 1D, E). This divergence between testis weight and sperm output is suggestive of spermatid loss during late spermiogenesis, wherein they contributed minimally to overall testis weight. Further, the more severe reduction in ESC compared to DSP is consistent with partial spermiation failure verified by the significant number of stage IX tubules with retained spermatids in *Ccdc112*^*KO/KO*^*mice*, 91.5%, compared to *Ccdc112*^*WT/WT*^, 20.2% (*p* < 0.001, Fig. 1H, I). Additionally, while both the number of round spermatids in stage VIII tubules (Fig. S2A) and apoptotic germ cells across all tubule stages (Fig. S2B) were comparable between genotypes, we observed elevated levels of sloughed cells with nuclear morphology consistent with round and elongated spermatids and to a lesser extend meiotic spermatocytes, and lymphocytes in the *Ccdc112*^*KO/KO*^ epididymides. The presence of such cells was rarely observed in *Ccdc112*^*WT/WT*^ mice (Fig. 1J).

**Fig. 2:**
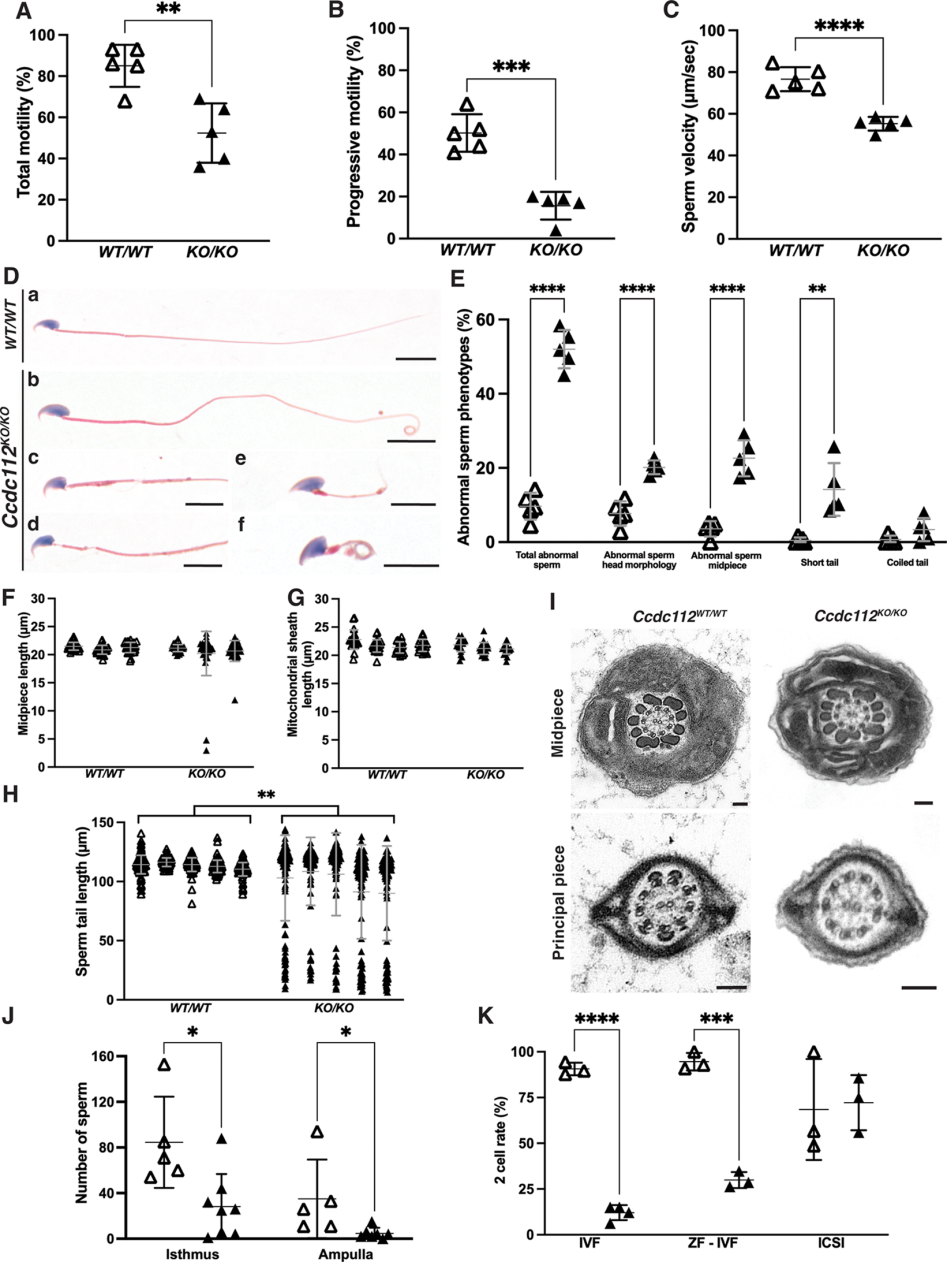
CCDC112 is essential for sperm tail structure and function and fertilization Percentage of motile (**A**) and progressively motile sperm (**B**) and average sperm velocity (VCL, curvilinear velocity; **C**) of cauda epididymal sperm from *Ccdc112*^*WT/WT*^ mice and *Ccdc112*^*KO/KO*^ mice (*n* = 5 mice/genotype). (**D**) Cauda epididymal sperm morphology as stained by hematoxylin and eosin. Abnormalities were observed in a majority of *Ccdc112*^*KO/KO*^ sperm, including abnormal sperm head morphology (**c**-**f**), mitochondrial sheath defects (**c**, **d**), short (**e**) and coiled (**f**) sperm tails. (**E**) Quantification of abnormal sperm morphology phenotypes in *Ccdc112*^*WT/WT*^ (white triangles) and *Ccdc112*^*KO/KO*^ males (black triangles) (*n* = 5/genotype). (**F**) Sperm midpiece length as stained by annulus marker, SEPT4; designating the junction of the putative mid- and principal pieces and as measured from the base of the sperm head to the annulus (*n* = 3/genotype). (**G**) Sperm mitochondrial sheath length as measured on hematoxylin and eosin stained sperm from the base of the sperm head to end of discernable sheath (*n* = 3–4/genotype). (**H**) Sperm tail length as measured from the sperm head base to the tail tip (*n* = 5/genotype). (**I**) Axoneme ultrastructure of cauda epididymal sperm from *Ccdc112*^*WT/WT*^ and *Ccdc112*^*KO/KO*^ males as assessed via transmission electron microscopy. (**J**) Quantification of *Ccdc112*^*WT/WT*^ (white triangles) and *Ccdc112*^*KO/KO*^ mouse sperm (black triangles) in the isthmus and ampulla region of the oviduct in the female reproductive tract (*n* ≥ 5/genotype). (**K**) Percentage of zonae pellucidae intact oocytes (IVF) and zonae pellucidae stripped oocytes (ZF – IVF) which developed to the two-cell stage after in vitro fertilization and percentage of oocytes which developed to the two-cell stage after intracytoplasmic sperm injection (ICSI) with *Ccdc112*^*WT/WT*^ and *Ccdc112*^*KO/KO*^ mouse sperm (*n* ≥ 3/genotype). Lines denote mean ± SD in A-C, E-H, J-K; * *P* < 0.05, ** *P* < 0.01, *** *P* < 0.001, **** *P* < 0.0001. Scale bars in D = 10 μm, I = 100 nm

Cauda epididymal sperm from *Ccdc112*^*KO/KO*^ males exhibited notably reduced total motility (85% in *Ccdc112*^*WT/WT*^ versus 52.4% in *Ccdc112*^*KO/KO*^; *p* < 0.01, Fig. 2A), progressive motility (50.2% versus 15.6%; *p* < 0.001, Fig. 2B) and speed (VCL, curvilinear velocity; 76.6 μm/s versus 56.7 μm/s; *p* < 0.0001, Fig. 2C) compared to sperm from wildtype males.

An examination of *Ccdc112*^*KO/KO*^ cauda epididymal sperm structure at a light microscopic level revealed that the majority were morphologically abnormal (52.02% abnormal in *Ccdc112*^*KO/KO*^ versus 9.66% in *Ccdc112*^*WT/WT*^; *p* < 0.0001, Fig. 2D, E). Defects included abnormal sperm head morphology (20.2% versus 7.7% *Ccdc112*^*WT/WT*^; *p* < 0.0001, Fig. 2D, E), midpiece structure abnormalities (22.6% versus 3.6%; *p* < 0.0001, Fig. 2Dc, d, E) and a higher incidence of short tails (14.2% versus 0.5%; *p* < 0.01, Fig. 2De, E). While sperm produced by *Ccdc112*^*KO/KO*^ males had normal midpiece and mitochondrial sheath lengths (Fig. 2F-G), average principal piece and total tail length were significantly reduced (*p* = 0.0089, Fig. 2H). Sperm fell into two major clusters – those with a normal sperm tail length and those where the tail was significantly shorter (*p* < 0.0001). Short tails are indicative of compromised axoneme/tail extension [70–72]noting that the mitochondrial sheath is loaded onto the sperm tail after ciliogenesis is complete [25, 26]. An assessment of the sperm axoneme structure via transmission electron microscopy revealed no difference in structure (Fig. 2I). In the majority of sperm, accessory structures including the outer dense fibers and fibrous sheath were present and superficially normal when viewed in a transverse plane.

Further analysis of sperm head morphology defects via testis section staining for α-tubulin as a marker of the manchette, a microtubule skirt-like structure essential for sperm head shaping, revealed comparable assembly, migration and disassembly of the manchette between genotypes (Fig. S3). Additional testing to determine the origin of the head morphology defects is required.

Given that 15.6% of sperm in *Ccdc112*^*KO/KO*^ exhibited progressive motility, fertilization remained possible. To test why this did not occur in vivo, we assessed the ability of sperm to ascend the female reproductive tract following natural mating (Fig. 2J). Compared to wildtype, 66% fewer sperm from *Ccdc112*^*KO/KO*^ males were present in the isthmus of the oviduct (*p* = 0.01) and 87% fewer in the ampulla (*p* = 0.04). To extend this analysis, we examined the consequences of CCDC112 loss on sperm function in in vitro fertilization, in the presence and absence of the zona pellucida, wherein the majority of physical barriers to fertilization were removed and intracytoplasmic sperm injection (ICSI) where all physical barriers were removed. Sperm from wildtype males achieved efficient fertilization with zonae pellucidae intact oocytes, leading to a two-cell development rate of 90.62% (*p* < 0.0001, Fig. 2J). By contrast, only 12.09% of zonae pellucidae intact oocytes were fertilized by sperm from *Ccdc112*^*KO/KO*^ males (*p* < 0.0001, Fig. 2K). A visual inspection revealed that the loss of CCDC112 impaired the ability of sperm to penetrate the zonae pellucidae (Fig. S4). Similarly, using zonae pellucidae stripped oocytes, wildtype male sperm achieved a fertilization rate of 94.59% compared to only 29.89% using *Ccdc112*^*KO/KO*^ mouse sperm (*p* < 0.0001, Fig. 2K). Finally, upon injection into the oocyte, two-cell development rate was comparable between genotypes (*p* = 0.85, Fig. 2K). CCDC112 is thus an essential requirement for mouse sperm to reach the site of fertilization in the female reproductive tract and to penetrate the outer vestments of the oocyte.

### CCDC112 regulates mitochondrial alignment and organization in the sperm midpiece

**Fig. 3:**
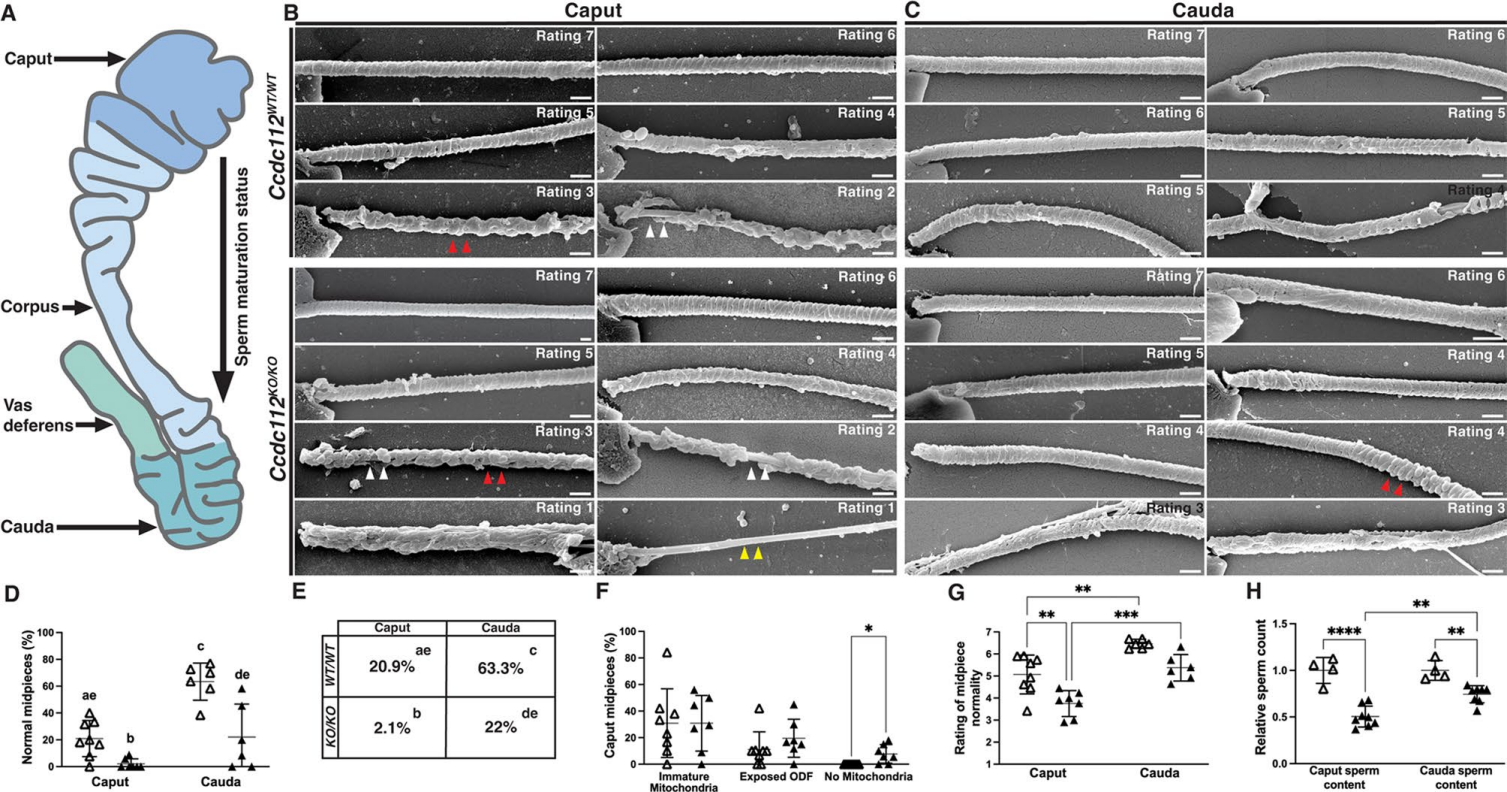
CCDC112 is critical for mitochondrial sheath formation (**A**) A schematic of the epididymis in mice. Once sperm are released from the testis they are transported to the epididymis for maturation, quality control, and storage. They first enter into the caput (head) epididymis before transiting through the corpus (body) towards the cauda (tail) before eventually exiting through the vas deferens upon ejaculation. Scanning electron micrography images of caput (**B**) and cauda (**C**) epididymal sperm midpieces from *Ccdc112*^*WT/WT*^ and *Ccdc112*^*KO/KO*^ mice. Midpieces were rated on a scale of 1 to 7, based on mitochondrial sheath abnormality severity. A score of 1 represents highly disorganized and abnormal mitochondrial sheath formation and a score of 7 represents highly organized mitochondrial alignment, i.e., normal mitochondrial sheath formation. No rating 1 sperm were seen in *Ccdc112*^*WT/WT*^ samples. Abnormalities included irregular alignment, morphology and size of mitochondria, immature mitochondria (red arrowheads), exposed outer dense fibers (white arrowheads; ODFs) and no mitochondria (yellow arrowheads). Percentage of normal caput and cauda midpieces (**D**-**E**) in *Ccdc112*^*WT/WT*^ (white triangles) and *Ccdc112*^*KO/KO*^ (black triangles) males (*n* ≥ 10 midpieces/animal and *n* ≥ 4/genotype). Differing lowercase letter designations denote significant differences between groups in D-E. Percentage of caput midpieces with immature ODF and/or no mitochondria (**F**), the rating of caput and cauda epididymal sperm midpiece normality (**G**), and the relative difference between caput sperm content and cauda sperm content (**H**) in *Ccdc112*^*WT/WT*^ and *Ccdc112*^*KO/KO*^ males (*n* ≥ 10 midpieces/animal and *n* ≥ 4/genotype). Lines denote mean ± SD in **D**, **F**-**H**; * *P* < 0.05, ** *P* < 0.01, *** *P* < 0.001, **** *P* < 0.0001. Scale bars in B-C = 0.5 μm

To further explore the consequences of CCDC112 loss on sperm morphology, we used scanning electron microscopy on plasma membrane stripped sperm from wildtype and Ccdc112^KO/KO^ mouse caput and cauda epididymides (Fig. 3A). First, we examined the organization of the mitochondrial and fibrous sheaths in wildtype sperm. The results revealed a previously unappreciated finding – that the quality of sperm mitochondrial sheath packing in wildtype sperm improves between the caput (proximal region) and the cauda (distal region) epididymis (Fig. 3), suggestive of a novel form of epididymal sperm maturation. A qualitative overview of sperm from the caput epididymis revealed that the majority of sperm contained mitochondrial defects, including misaligned, incorrectly oriented, and morphologically abnormal (inconsistent shape and size) mitochondria, immature (spherical and/or crescent shaped) mitochondria, absent mitochondria, and/or exposed outer dense fibers. By contrast, defects observed in cauda epididymal sperm midpieces, where they existed, were milder in nature with the most severe abnormalities not being present (Fig. 3B, F). A similar but more severe range of defects were observed in sperm from *Ccdc112*^*KO/KO*^ males with additional defects included the complete absence of mitochondria along sections of or the whole midpiece (Fig. 3F).

To quantify these defects, sperm midpiece structure was scored on a normality scale (ranging from 1 – highly abnormal to 7 – high quality [normal]) between epididymal regions and genotypes. For wildtype caput epididymis sperm, 20.9% of sperm were classified as high quality (rating 7) compared to 63.3% of sperm from the cauda region, thus highlighting the improvement in sperm structural quality occurring during epididymal transit (*p* < 0.0001, Fig. 3D-E). For *Ccdc112*^*KO/KO*^ mice, only 2.1% of caput epididymal sperm were high quality (rating 7), in comparison to 22% of cauda sperm (*p* = 0.028, Fig. 3D-E). A comparison between genotypes and epididymis regions revealed that CCDC112 function is required to produce normal numbers of high-quality cauda epididymal sperm, with 63.3% being present in the *Ccdc112*^*WT/WT*^ cauda epididymis versus 22% in *Ccdc112*^*KO/KO*^ (*p* < 0.0001; Fig. 3D-E. Only 20.9% of caput sperm were classified as normal (rating 7) in *Ccdc112*^*WT/WT*^ and 2.1% in *Ccdc112*^*KO/KO*^ (*p* = 0.026; Fig. 3D-E), noting the additional significant reduction in total epididymal sperm numbers in *Ccdc112*^*KO/KO*^ males.

Further, of the defects observed, their overall severity was significantly greater in *Ccdc112*^*KO/KO*^ mouse sperm compared to wildtype, in both the caput and cauda epididymis. For *Ccdc112*^*KO/KO*^ mouse caput epididymal sperm, a significantly lower average midpiece normality rating of 3.75 was measured, compared to 5.07 in wildtype (*p* = 0.0033, Fig. 3G). For cauda epididymal sperm, an average normality rating of 5.38 was measured in *Ccdc112*^*KO/KO*^ mouse sperm compared to 6.46 in wildtype (*p* = 0.036, Fig. 3G). Notably, regardless of genotype, cauda epididymal sperm had higher quality midpieces than those found in the caput, and accordingly, a significant increase in normality rating (*Ccdc112*^*WT/WT*^– 5.07 in caput to 6.46 in cauda, *p* = 0.0029; *Ccdc112*^*KO/KO*^– 3.75 in caput to 5.38 to cauda, *p* = 0.0008, Fig. 3G).

Finally, we measured sperm numbers in the caput and cauda epididymis segments in wildtype and *Ccdc112*^*KO/KO*^ mice. As above, a 13% decrease in DSP was detected in the *Ccdc112*^*KO/KO*^ mice testis compared to wildtype (*p* < 0.05, Fig. 1D). Significantly, in *Ccdc112*^*KO/KO*^ individuals, a 54% decrease in sperm number was measured in the caput epididymis relative to wildtype (*p* < 0.0001, Fig. 3H), and a 35% decrease in sperm number in the cauda epididymis (*p* = 0.0053, Fig. 3H) i.e. cumulatively between the testis, caput and cauda, sperm numbers in the *Ccdc112*^*KO/KO*^ epididymis are significantly reduced compared to wildtype.

Collectively, these data reveal that during sperm epididymal transit (1) midpiece maturation continues and (2) morphologically abnormal sperm are selectively removed, potentially as a means of quality control. As an illustration of the later, sperm with midpieces partially or fully devoid of mitochondria in the caput epididymis were absent from the cauda. Given that sperm mitochondrial acquisition from external sources is unlikely in the epididymis, the absence of such sperm in the cauda epididymis is strongly indicative of their removal from the population. Equally, the presence of only 2.1% normal (rating 7) *Ccdc112*^*KO/KO*^ mouse sperm in the caput compared to 22% normal sperm in cauda is conclusive of the ongoing sperm midpiece maturation during epididymal transit. Despite this quality control mechanism, the majority of cauda epididymal sperm from *Ccdc112*^*KO/KO*^ males were abnormal, i.e. 22% of null sperm were morphological normal and only 15.6% were capable of progressive motility, compared to 63.3% and 50.2%, respectively, in sperm from wildtype. No sperm from *Ccdc112*^*KO/KO*^ males could achieve fertility in vivo.

### CCDC112 is critical for flagella kinematics

**Fig. 4:**
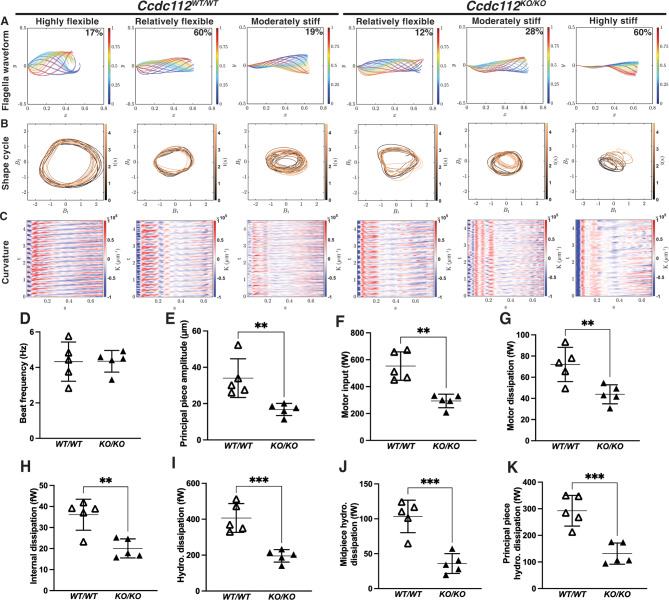
Sperm tail beating patterns and energetics are critically dependent on CCDC112 (**A**) Representative sperm flagella waveform in sperm from *Ccdc112*^*WT/WT*^ and *Ccdc112*^*KO/KO*^ mice over the proximal most 60 μm of the sperm tail. Colored curves represent the non-dimensional time scale, where the beginning of the beat cycle is blue in the waveform and the end is in red. ‘y’ denotes the normalized amplitude and ‘x’ denotes the normalized length of the sperm tail where the y-intercept of 0 represents the sperm head. Percentage of sperm per genotype displaying each beat cycle type is denoted in the top right corner of each graph. (**B**) Representative shape cycles of the same sperm population in (**A**), as visualized by plotting the two dominant shape modes coefficients (B_1_ and B_2_) against each other over time (*n* = 5 animals/genotype and 8–15 sperm/animal). (**C**) Representative flagellum curvature plots, where the red and blue waves denote the direction of the flagellar bend above or below the sperm head hook and the intensity of the color denotes smaller radius of curvature (*n* = 5 animals/genotype and 8–15 sperm/animal). ‘T’ denotes time (seconds), and ‘s’ denotes arclength (µm/100) where 0 represents the sperm head. Beat frequency (**D**), principal piece amplitude (**E**), motor input (**F**), motor dissipation (**G**), internal dissipation (**H**), hydrodynamic dissipation (**I**), midpiece hydrodynamic dissipation (**J**) and principal piece hydrodynamic dissipation (**K**). Lines denote mean ± SD in D-K; ** P < 0.01, *** P < 0.001

To define how CCDC112 loss affected sperm tail waveform, high speed imaging analysis was performed on cauda epididymal sperm [61]. As shown in Supplementary Video 1 and Fig. 4, wildtype mouse sperm flagellum movement was flexible throughout all tail segments (Fig. 4A) and highly repetitive (Fig. 4B). By contrast, *Ccdc112*^*KO/KO*^ mouse sperm flagellum movement was rigid, notably in the midpiece (Supplementary Video 2; Fig. 4). An analysis of the flagella waveform revealed that the majority of sperm from *Ccdc112*^*KO/KO*^ males possessed a stiff midpiece (Fig. 4A). Specifically, 17% of motile sperm from *Ccdc112*^*WT/WT*^ mice had a highly flexible waveform wherein midpiece amplitude ranged between 27 and 39 μm (Fig. 4A), 60% were relatively flexible with a midpiece amplitude ranging 16–27 μm (Fig. 4A), 19% were moderately stiff with an amplitude of 6–15 μm (Fig. 4A), and 4% were highly stiff with a midpiece amplitude ranging between 0.6 and 6 μm (data not shown). By contrast for *Ccdc112*^*KO/KO*^ males, there were no highly flexible sperm, 12% of midpieces were relatively flexible (Fig. 4A), 28% were moderately stiff (Fig. 4A) and the majority, 60%, were highly stiff (Fig. 4A). Collectively, 88% of sperm midpieces from *Ccdc112*^*KO/KO*^ males were classified as stiff compared to 22% in wildtype. Correspondingly, the amplitude in the principal piece was significantly decreased (16.79 μm versus 34.04 μm; *p* = 0.0086, Fig. 4E) in *Ccdc112*^*KO/KO*^ mouse sperm compared wildtype. The tail beating frequency (i.e., the number of beating cycles per second) was comparable between genotypes (Fig. 4D).

Next, to analyze waveform periodicity over time, sperm flagella shape cycle was assessed. Here, a circular shape cycle is indicative of highly reproducible and periodic waveform beating behavior with a shape cycle displaying a more imperfect circle indicating a more variable beating behavior. In wildtype mouse sperm, the majority of the shape cycles were uniform circles with some irregularity being observed in sperm with stiffened midpieces. Comparatively, *Ccdc112*^*KO/KO*^ mouse sperm shape cycles presented most commonly with highly irregular, non-uniform circles indicative of significant variability in waveform between beats.

To illustrate the role of CCDC112 in the flagella, particularly for midpiece flexibility over multiple beat cycles, we plotted sperm tail curvature along the tail length over a period of 4.5 s (Fig. 4C). As shown for wildtype sperm, the alternating blue and red strips in the curvature kymograph indicate the wave propagation and formation along the flagellum is highly periodic and reproducible. By comparison, sperm from *Ccdc112*^*KO/KO*^ males, particularly those with a highly stiffened midpiece, displayed considerably variable and disturbed flagella wave propagation and formation over both space and time (Fig. 4C). Collectively, these data suggest that CCDC112 contributes to the spatial organization of the mitochondrial sheath, thus affecting sperm tail flexibility, particularly in the midpiece.

At a subcellular level, sperm flagellum movement is generated by forces produced from dynein motors within the axoneme. Specifically, the power exerted from the flagellum dynein motors to axonemal microtubules is termed the motor input [62]. Our results suggest that motor input was significantly lower in *Ccdc112*^*KO/KO*^ mouse sperm in comparison to wildtype (293 fW versus 561 fW; *p* = 0.0011, Fig. 4F), indicating lower rates of energy production. Next, internal, and external dissipated power were assessed. Internal energy dissipation in the flagellum involves both motor dissipation, originating from the work done by other flagellar structures on dynein motors, and internal dissipation, arising from friction between cross-sectional planes within the flagellum. External energy dissipation, known as hydrodynamic dissipation, results from the friction between the flagellum and the surrounding fluid. Our data showed that all power dissipations were significantly reduced in *Ccdc112*^*KO/KO*^ mice (*p* = 0.0093, Fig. 4G; *p* = 0.0033, Fig. 4H and *p* = 0.0007, Fig. 4I). Moreover, total hydrodynamic dissipation can be further divided into contributions from the midpiece and principal piece, both of which were significantly lower in sperm from *Ccdc112*^*KO/KO*^ males compared to wildtype (*p* = 0.005 and *p* = 0.0009, respectively, Fig. 4J-K).

### CCDC112 is required for optimal sperm metabolism

**Fig. 5:**
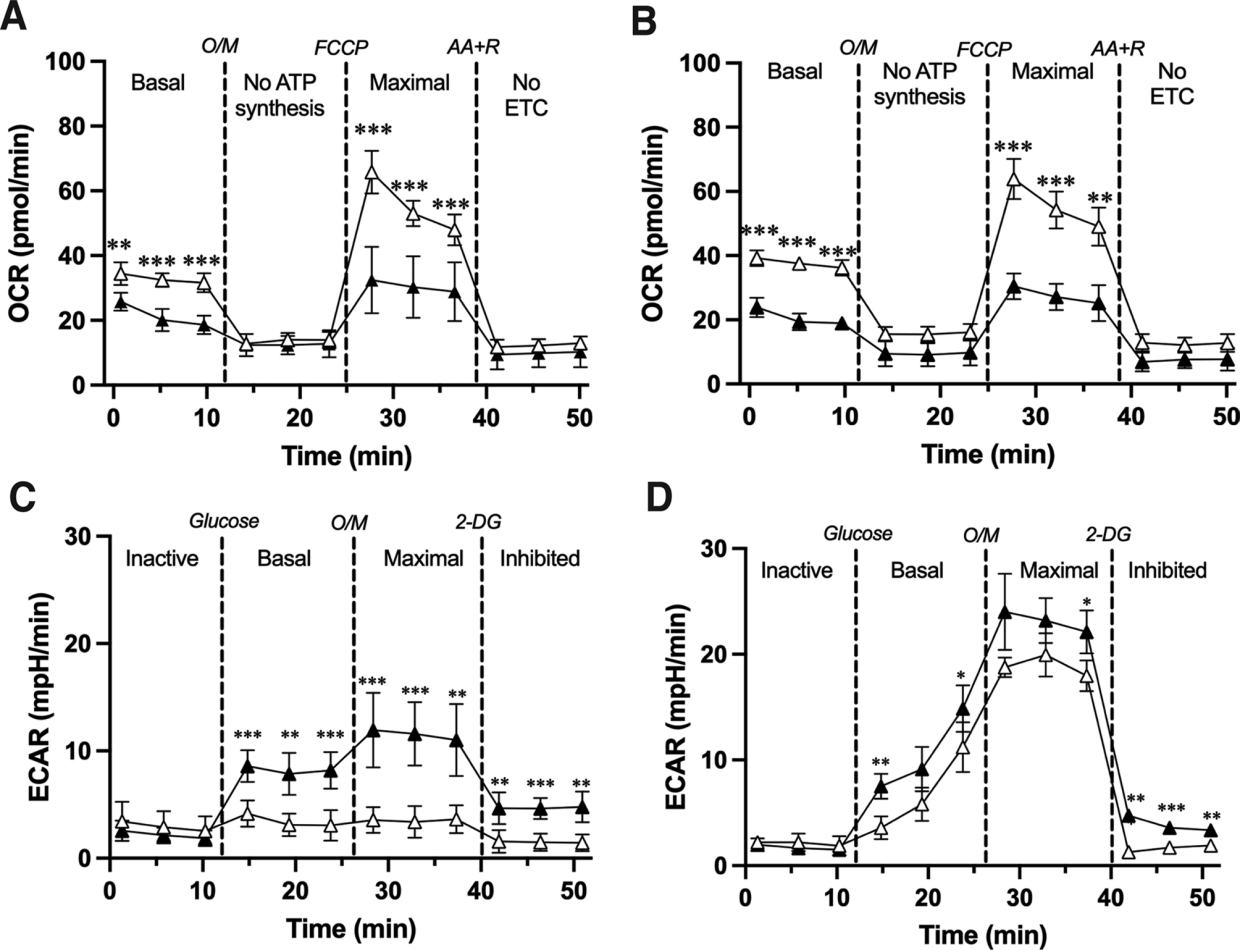
CCDC112 is essential for normal sperm energy production Mitochondria stress test assay on non-capacitated (**A**) and capacitated (**B**) *Ccdc112*^*WT/WT*^ (white triangles) and *Ccdc112*^*KO/KO*^ mouse sperm (black triangles) (*n* = 3 mice/genotype). Glycolytic flux Seahorse assay on non-capacitated (**C**) and capacitated (**D**) *Ccdc112*^*WT/WT*^ and *Ccdc112*^*KO/KO*^ mouse sperm (*n* = 3 mice/genotype). Note the difference in the Y axis scale between mitochondrial stress test and glycolytic assays. OCR = oxygen consumption rate; ECAR = extracellular acidification rate; O/M = oligomycin; AA + R = antimycin A and rotenone; 2-DG = 2-Deoxy-D-Glucose. Lines denote mean ± SD; * *P* < 0.05, ** *P* < 0.01, *** *P* < 0.001

Ultimately sperm motility is driven by ATP hydrolysis by the dynein arms of the axoneme leading to microtubule sliding [73, 74]. Within sperm, ATP production occurs via two dominant processes, oxidative phosphorylation in the midpiece mitochondria, and glycolysis via enzymes primarily anchored to the fibrous sheath in the principal piece [75]. To test the effect of CCDC112 loss and poor mitochondrial packing on mitochondrial respiration capacity (oxidative phosphorylation), we used the Seahorse mitochondrial stress test assay on both non-capacitated and capacitated sperm in wildtype versus *Ccdc112*^*KO/KO*^ mice (Fig. 5). Our data on wildtype mouse sperm showed no significant difference between mitochondrial respiration rates in non-capacitated versus capacitated sperm (Fig. S5A). Similarly, in the absence of CCDC112 no significant difference between non-capacitated versus capacitated sperm was measured (Fig. S5B). However, when compared between genotypes, oxygen consumption data revealed that, in non-capacitated sperm, basal and maximal mitochondria respiration of sperm from *Ccdc112*^*KO/KO*^ males was significantly impaired relative to wildtype (34% basal and 45% maximal rate reduction; *p* < 0.01, Fig. 5A). This difference was exacerbated in capacitated sperm, where basal and maximal mitochondria respiration rates were 45% lower for basal and 50% for maximal (*p* < 0.01, Fig. 5B) in sperm from *Ccdc112*^*KO/KO*^ males compared to those from wildtype siblings. CCDC112 function is thus required for optimal oxidative phosphorylation in sperm. We hypothesize this is via its essential role in mitochondrial sheath formation.

As reported previously, capacitation in mouse sperm is associated with a notable increase in glycolysis as measured using a glycolytic flux Seahorse assay [8]. Specifically, we observed a 99.21% and 434.84% increase in the basal and maximal glycolysis activity, respectively, in capacitated versus non-capacitated sperm for wildtype males (*p* < 0.001 and *p* < 0.0001, respectively, Fig. S4C-D). For *Ccdc112*^*KO/KO*^ mouse sperm we observed a 27.98% and 100.7% increase in basal and maximal glycolysis activity, respectively, in capacitated versus non-capacitated sperm (*p* < 0.001, Fig. S4C-D). When compared between genotypes, and by contrast to the data for oxidative phosphorylation, sperm from *Ccdc112*^*KO/KO*^ males exhibited significantly increased glycolysis activity compared to wildtype (Fig. 5C-D). Specifically, in the absence of CCDC112, the basal and maximal glycolysis capacity in non-capacitated sperm was increased by 138% and 226%, respectively, compared to wildtype (*p* < 0.01, Fig. 5C) suggesting a degree of metabolic reprogramming in sperm lacking CCDC112. While in capacitated *Ccdc112*^*KO/KO*^ mouse sperm, basal and maximal glycolytic activity was increased by 53% and 22% above wildtype levels (*p* < 0.05, Fig. 5D). As expected, when a competitive inhibitor, 2-deoxyglucose was added, sperm from wildtype mice returned to inactive levels, however, *Ccdc112*^*KO/KO*^ mouse sperm maintained a significantly higher glycolytic capacity, independent of capacitation status, confirming the up-regulation of glycolysis in sperm in the absence of CCDC112.

Finally, we assessed whether mitochondrial sheath structure abnormalities affected sperm mitochondrial membrane potential using the JC-1 probe. When mitochondrial membrane potential is high, JC-1 accumulates in mitochondria and emits a red fluorescence. By contrast, JC-1 emits a green fluorescence when potential is low. Mitochondria membrane potential was unchanged between *Ccdc112*^*WT/WT*^ and *Ccdc112*^*KO/KO*^ mouse sperm (*p* = 0.13, Fig. S6), suggesting sperm mitochondria were functional at a basic level.

Collectively, these data reveal that in the absence of CCDC112, ATP production in non-capacitated and capacitated sperm is significantly impaired. Membrane potential data suggests that the decrease is due to mitochondrial sheath structural defects. We cannot, however, preclude the possibility that the abnormal mitochondrial packing disrupts mitochondria-mitochondria linking and synergistic effects on mitochondria function. While mitochondrial fusion has not been observed, or explored, in sperm, inter-mitochondrial linker structures between adjacent mitochondria have been identified [76]. The role of these linkers remains unknown, however, their association with mitochondrial cristae suggest the potential to facilitate electrochemical coupling, energy flow and/or communication between mitochondria [76, 77]; processes which could all be disrupted in the absence of CCDC112.

### CCDC112 is a component of the distal appendages of the mother centriole in somatic cells

**Fig. 6:**
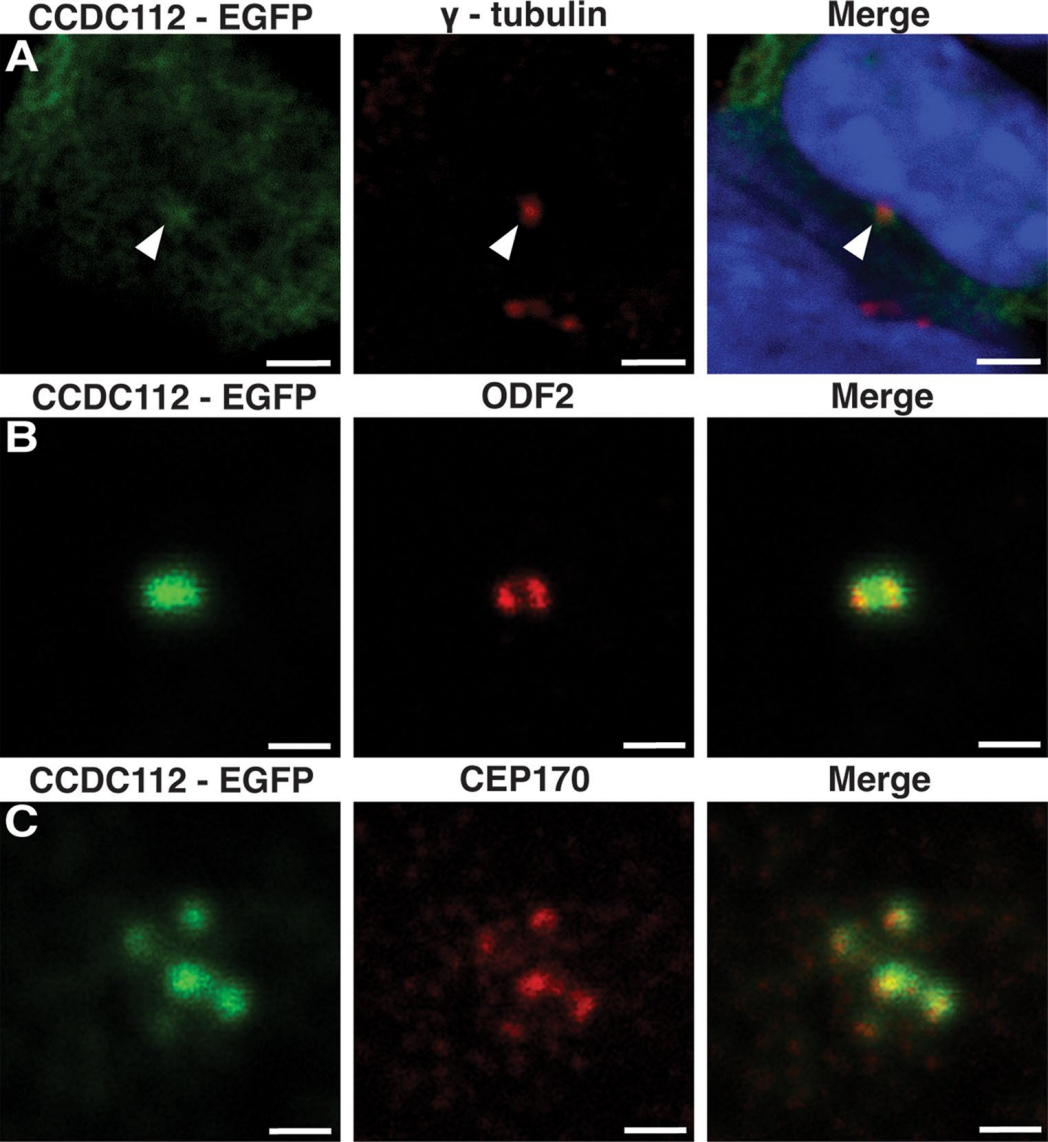
CCDC112 is a component of the distal appendages of the mother centriole Immunofluorescence staining of somatic IMCD-3 cells induced to produce primary cilia via serum starvation expressing e-GFP tagged CCDC112 (green). CCDC112 localized to the centrosome (as marked by γ-tubulin; red) (**A**). Staining of the distal and subdistal appendages as marked by ODF2 (red) refined localization to the mother centriole (**B**). Further staining localized CCDC112 specifically to the distal appendages (as marked by CEP170; red) of the mother centriole in interphase cells (**C**). Blue represents nuclei labelled by DAPI. Scale bars in A = 3 μm and in B-C = 1 μm

Given previous proteomics data suggesting that CCDC112 is a centriolar satellite protein in RPE-1 cells [47]we aimed to resolve CCDC112 localization in somatic ciliated IMCD-3 cells by expressing exogenous eGFP-tagged CCDC112. CCDC112 localization was observed at the centrosome as marked by γ-tubulin (Fig. 6A). To refine localization, cells were co-stained with ODF2, a marker of the distal and subdistal appendages at the proximal end of the mother centriole [78]. STED microscopy revealed that CCDC112 co-localized with ODF2 in the mother centriole (Fig. 6B). These findings were validated by co-staining with CEP164 (data not shown), a specific marker of subdistal appendages [79] and CEP170, a specific marker of distal appendages [80] revealing the localization of CCDC112 solely to the distal appendages, and not subdistal appendages, in interphase cells (Fig. 6C).

Collectively, such a localization and the reduced sperm tail length in a sub-population of sperm in mature *Ccdc112*^*KO/KO*^ mouse sperm, indicate that CCDC112 is required for both normal motile cilia/flagella production by haploid germ cells, and for the assembly of the mitochondrial sheath.

## Discussion

The mitochondrial sheath is the defining structure of the sperm midpiece, providing structural support and hypothesized to generate energy necessary for multiple aspects of flagella movement [3, 4, 6]. Importantly, abnormalities to this structure have been associated with human male infertility [31, 81–83]. While many of the key steps of sperm midpiece formation have been described at a cytological level [31]the molecular processes required for its assembly and function remain poorly understood. Herein, we show that CCDC112 is essential for both processes. Specifically, we have identified a previously unrecognized process of mitochondrial sheath maturation that occurs during epididymal sperm maturation. We demonstrate a critical role for CCDC112 in mitochondrial morphogenesis and remodeling during mitochondrial sheath formation in the testis. We show that such remodeling and the associated optimization of ATP production is critical to the manifestation of a highly effective sperm flagella waveform and mechanical power and, in turn, the capacity of sperm to reach and fertilize oocytes within the female reproductive tract.

Our data also establish the novel process of mitochondrial sheath maturation as one of the key events in the sperm epididymal maturation process. While other types of sperm maturation within the epididymis have been documented, including biochemical and plasma membrane modifications [84–87], the acquisition of potential for sperm motility [88] and the transfer of RNA cargoes, proteins and lipids via sperm epididymosomes (epididymis specific extracellular vesicles) interactions [89], no study to date has described complex sperm organelle structural modifications during this period. Our data show that at the time of spermiation, the majority of spermatozoa are structurally immature, and that maturation continues as sperm transit through the epididymis from the caput to the cauda. Such maturation includes elongation/morphogenesis of mitochondria into a rod-like form and/or further compaction of mitochondria within the sheath. In agreement, we observed an increase in sperm population midpiece quality in the distal epididymis. Simultaneously, our data evidence that poor-quality sperm with highly abnormal midpiece phenotypes e.g. absent mitochondria, are selectively removed from the epididymis during transit.

While the specific removal of sperm with structural defects is a novel observation, the selective removal of abnormal sperm is consistent with a report from Glover [90]who observed a higher rate of decapitated and dead sperm in the proximal corpus compared to the cauda in cat, dog, goat and bovine epididymides. While the exact method of removal remains poorly defined, data from numerous mammalian species, including mice, suggest that misshapen sperm are captured in a dense matrix formed via the release and subsequent merging of aposomes released from the epididymal epithelium [91–93]. The entanglement of abnormal sperm separates them from viable sperm, allowing for their disintegration and dissolution by epididymosomes, present within aposomes [91]. Further, while instances of sperm phagocytosis or spermiophagy have been reported in mammals [94–96]this is a rare occurrence and is not the primary quality control mechanism mediating the removal of abnormal sperm [97, 98]. Data presented here also suggest that while this quality control process can scale to some degree, as indicated by the 35% reduction in *Ccdc112*^*KO/KO*^ cauda epididymal sperm count compared to wildtype, at some point it is overwhelmed, leading to the presence of residual abnormal sperm in the ejaculate. This selective removal of abnormal sperm sits ‘on top of’ an analogous process that occurs in the testis that can trigger spermiation failure [99, 100].

**Fig. 7:**
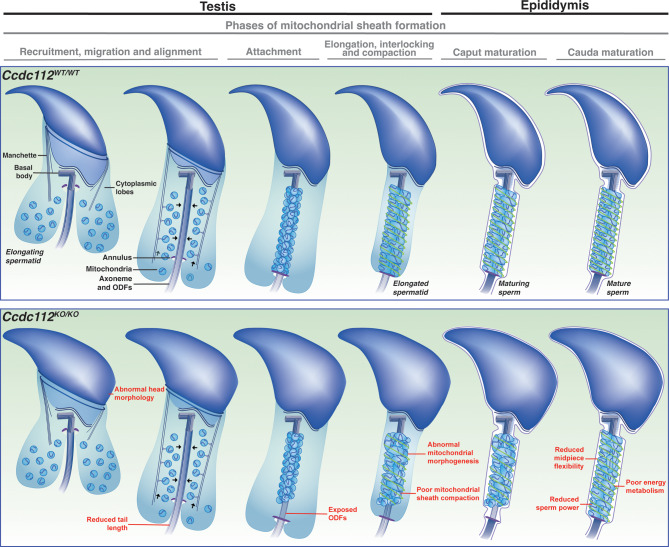
Summary schematic of sperm formation in the absence of CCDC112 In *Ccdc112*^*KO/KO*^ mice, sperm commonly possessed abnormal mitochondrial sheath structure with overall irregular mitochondrial morphology and poor mitochondrial elongation, staggering and compaction during sheath formation. A subset of sperm also possessed exposed outer dense fibers (ODFs) and immature mitochondria within the sheath, abnormal head morphology and shortened tails. As a result, sperm exhibited poor energy generation, reduced tail flexibility, particularly in the midpiece, and reduced sperm power. Image modified from [73]

As defined above, mitochondrial loading and remodeling within the midpiece is a multi-step process that initiates in the final steps of spermatogenesis in the testis. In the absence of CCDC112, the recruitment of mitochondria from the cytoplasm appears largely normal (Fig. 7). The translocation and attachment of mitochondria to the outer dense fibers also appears mostly unaffected, with the exception of a small percentage of cells where mitochondria failed to load onto, or attach to, the outer dense fibers. By contrast, the morphogenesis of mitochondria from spherical to crescent then to rod shaped and the staggering and compaction of mitochondria into the distinct double helical structure of the sheath, was impaired in the absence of CCDC112. As a result of these defects paired with an underlying deficit in ATP production and insufficient energy utilization, sperm presented with a midpiece with restrained flexibility. Whether the stiffness is a consequence of abnormal structures and/or the unavailability of ATP remains to be determined. Consequently, in the absence of CCDC112, the flagellar waveform was unable to produce sufficient power to sustain progressive motility to reach the upper oviduct and fertilize oocytes. Due to experimental limitations, we were unable to directly examine elongated spermatid mitochondrial sheath structure directly, instead assessing mitochondrial sheath structure of caput and cauda epididymal sperm. Specifically, our attempts to analysis midpiece structure in testicular elongated spermatids / sperm suggest that mitochondria are tenuously attached to the outer dense fibers in both wildtype and *Ccdc112* null mice, leading to detachment during the purification process. As such we were unable to reliably analyze the consequences of CCDC112 loss beyond that shown using SEM and TEM methods which clearly reveal a role for CCDC112 in regulating mitochondrial sheath formation in the testis.

Our data also support a facilitative role for CCDC112 as a component of the distal appendages of the basal body, in the development of the sperm axoneme and thus sperm tail length. In mouse spermatogenesis the basal body docks to both the sperm nucleus and plasma membrane in step 2/3 [101]. This ultimately forms a separate lobe, the ciliary lobe, which is isolated from the remainder of the cytoplasm (the cytoplasmic lobe) [2]. Subsequently, the axoneme extends from the basal body into the ciliary lobe to form the core of the sperm tail [2]. The localization of CCDC112 to the distal appendages in IMCD-3 cells suggests it functions at a similar location in round spermatids, where it may ultimately contribute to controlling protein entry into the ciliary compartment and thus sperm tail growth. The hypothesis is supported by the presence of a distinct population of sperm with short tails within the cauda epididymis from *Ccdc112*^*KO/KO*^ males but remains to be formally tested. Whether such abnormalities in either sperm tail length and/or mitochondrial sheath structure are the cause of abnormal retention of elongated spermatozoa in the testis also remains to be tested.

In summary, we propose that CCDC112 executes its function via two biological processes. First, CCDC112 may function at the basal body to facilitate early steps of tail development by contributing to the efficient regulation of protein entry, including proteins that ultimately determine tail length and mitochondrial sheath formation, into the sperm tail [102]. Second, CCDC112 contributes to tail function through a role in mitochondrial sheath formation and ultimately optimizing ATP production. This later role may also be direct or could be indirect, including as a scaffold involved in mitochondrial movement or stability. Collectively, this study adds to the field’s knowledge of how functional sperm are produced and the causes of male infertility. We reveal a novel form of epididymal sperm maturation, a novel mechanism of mitochondria-mitochondria interactions of potential relevance to multiple tissues, and the complex relationship between energy production and sperm tail kinematics.

## Electronic supplementary material

Below is the link to the electronic supplementary material.

Supplementary Material 1

Supplementary Material 2

Supplementary Material 3

Supplementary Material 4

Supplementary Material 5

Supplementary Material 6

Supplementary Material 7

Supplementary Material 8

Supplementary Material 9

## Data Availability

No datasets were generated or analysed during the current study.

## Abbreviations

CASA: Computer assisted semen analysis
CCDC112: Coiled coil domain containing 112
DAB: Diaminobenzidine
DSP: Daily sperm production
ESC: Epididymal sperm content
hCG: Human chorionic gonadotrophin
ICSI: Intracytoplasmic sperm injection
IVF: In vitro fertilization
ODF: Outer dense fibers
qPCR: Quantitative polymerase chain reaction
