# Supplementary figures and images for: A novel mechanism of sperm midpiece epididymal maturation and the role of CCDC112 in sperm midpiece formation and establishing an optimal flagella waveform
Source: Cell Commun Signal. 2025 Jul 1;23:319. doi: 10.1186/s12964-025-02320-x (PMC12218091; doi:10.1186/s12964-025-02320-x)

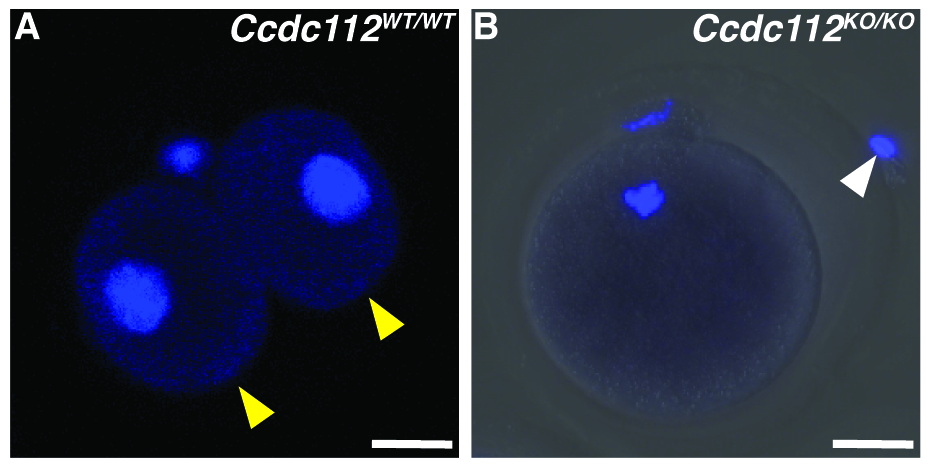

Supplement: Supplementary file 1 — Supplementary Material 1 [file 12964_2025_2320_MOESM1_ESM.tif]

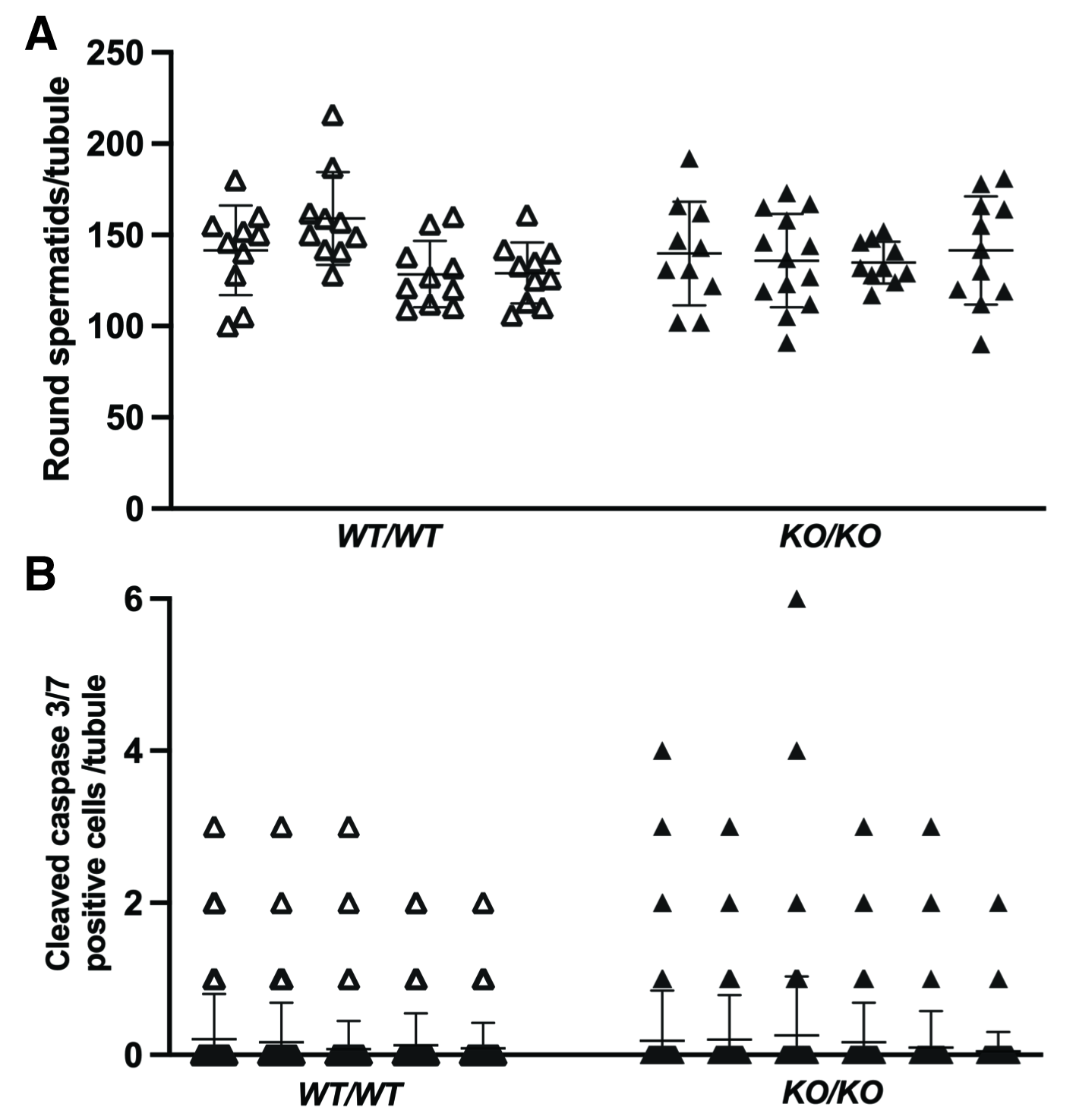

Supplement: Supplementary file 2 — Supplementary Material 2 [file 12964_2025_2320_MOESM2_ESM.tif]

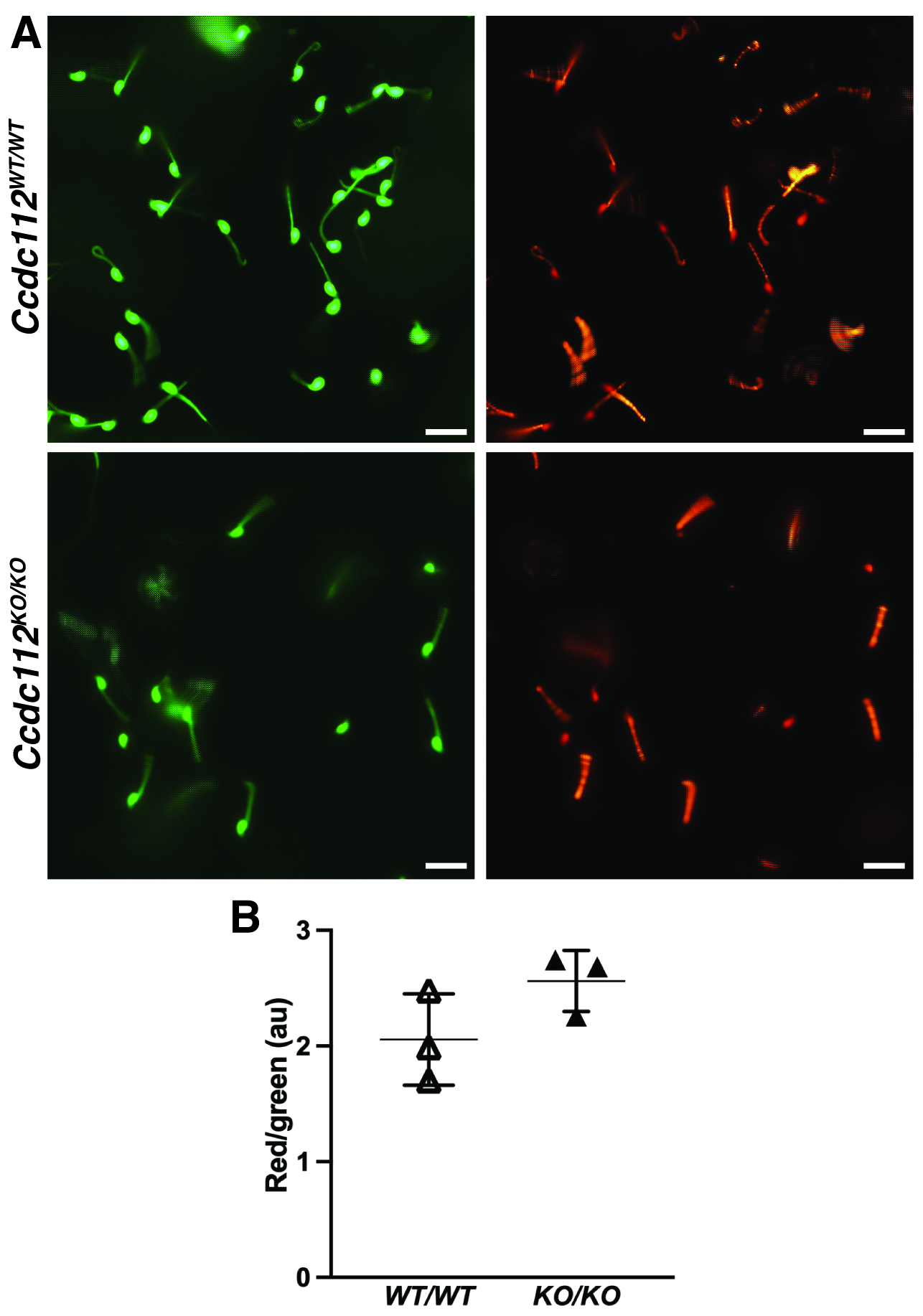

Supplement: Supplementary file 3 — Supplementary Material 3 [file 12964_2025_2320_MOESM3_ESM.tif]

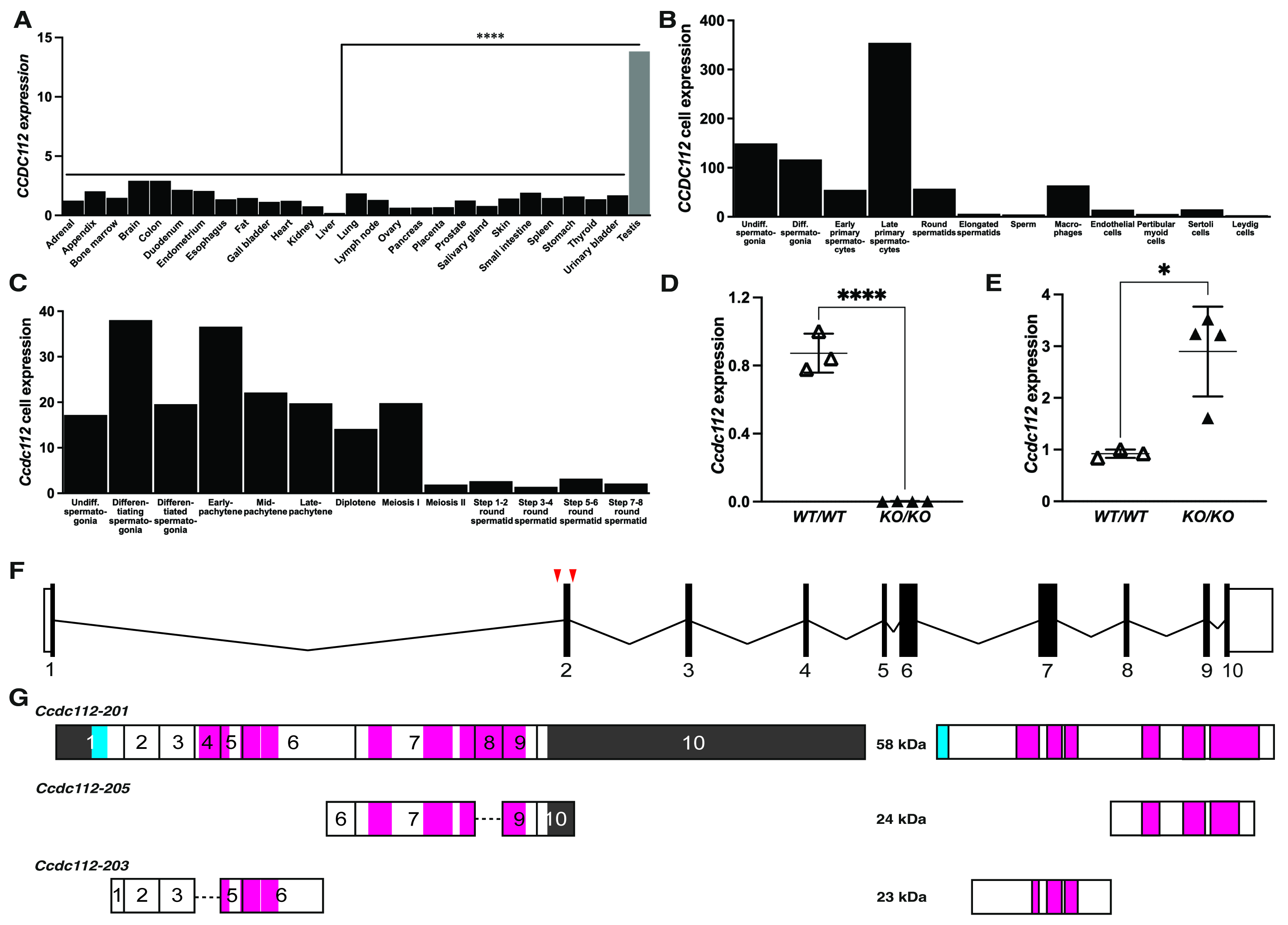

Supplement: Supplementary file 4 — Supplementary Material 4 [file 12964_2025_2320_MOESM4_ESM.tif]

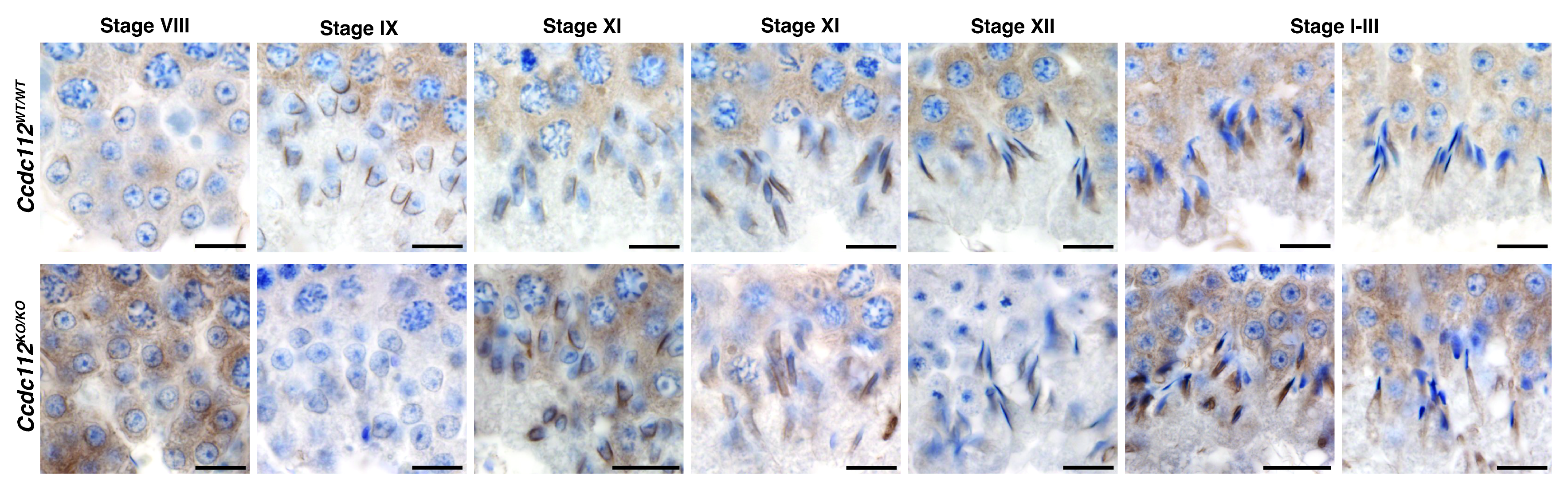

Supplement: Supplementary file 5 — Supplementary Material 5 [file 12964_2025_2320_MOESM5_ESM.tif]

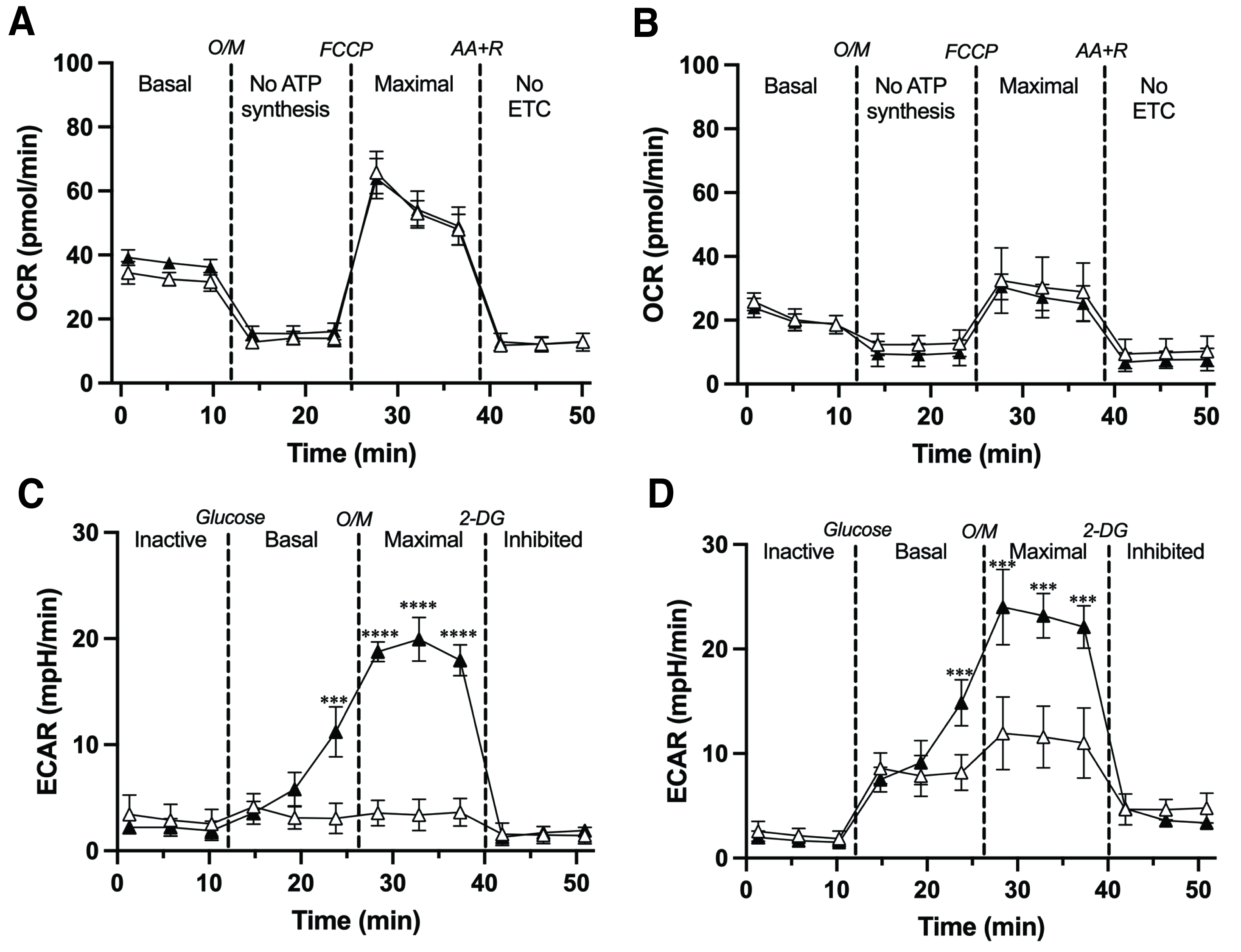

Supplement: Supplementary file 7 — Supplementary Material 7 [file 12964_2025_2320_MOESM7_ESM.tif]
