# Supplementary material for: A novel mechanism of sperm midpiece epididymal maturation and the role of CCDC112 in sperm midpiece formation and establishing an optimal flagella waveform
Source: Cell Commun Signal. 2025 Jul 1;23:319. doi: 10.1186/s12964-025-02320-x (PMC12218091; doi:10.1186/s12964-025-02320-x)
Supplement: Supplementary file 9 — Supplementary Material 9 [file 12964_2025_2320_MOESM9_ESM.docx]

**Supplementary data**


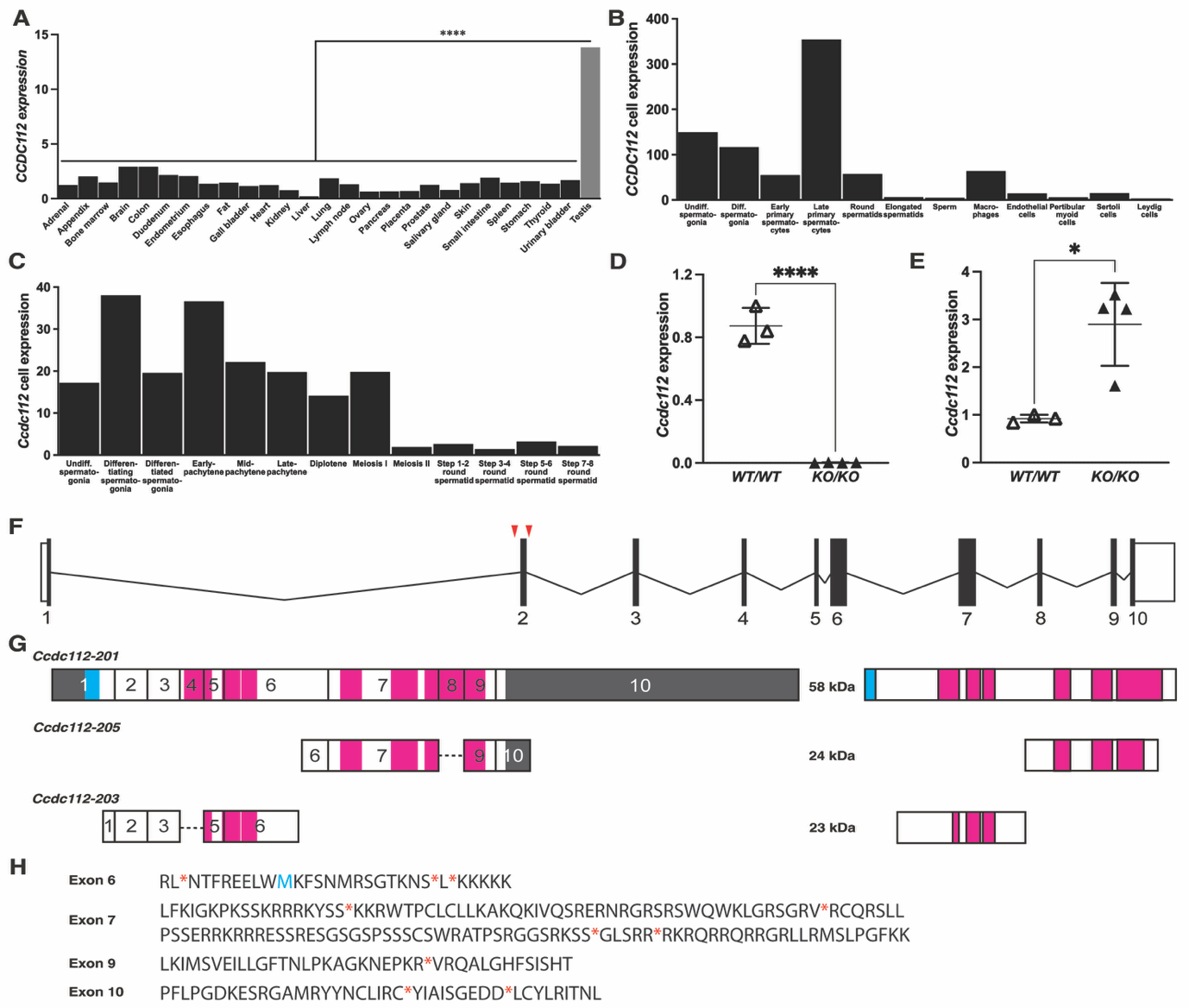


**Fig. S1. *Ccdc112* loss-of-function mouse model and expression in male germ cells**

*Ccdc112* expression in human organs (A) and human (B) and mouse (C) testis cell types as determined by single cell sequencing data ^51–53^. (D) *Ccdc112-201 and Ccdc112-203 transcript* expression in wildtype and loss-of -function testes, as measured by qPCR using primers located in exons 4/5 and 6. (E) *Ccdc112-201 and Ccdc112-205* transcript expression in wildtype and loss-of-function testes, as measured by qPCR with primers located in exons 7 and 9. Mouse *Ccdc112* exon map (F), including the predicted transcripts and their domains, and the corresponding proteins encoded (G). Signaling protein and coiled-coil domains are shown in blue and pink, respectively, while untranslated regions are shown in grey. Red arrowheads in (F) indicate guide RNA target regions used to excise exon 2 in the *Ccdc112^KO^*^/^*^KO^* mouse line. (H) Protein sequence of the *Ccdc112-205* transcript with blue denoting the start codon and red denoting subsequent stop codons. Lines denote mean ± SD; * P < 0.05, **** P < 0.0001.


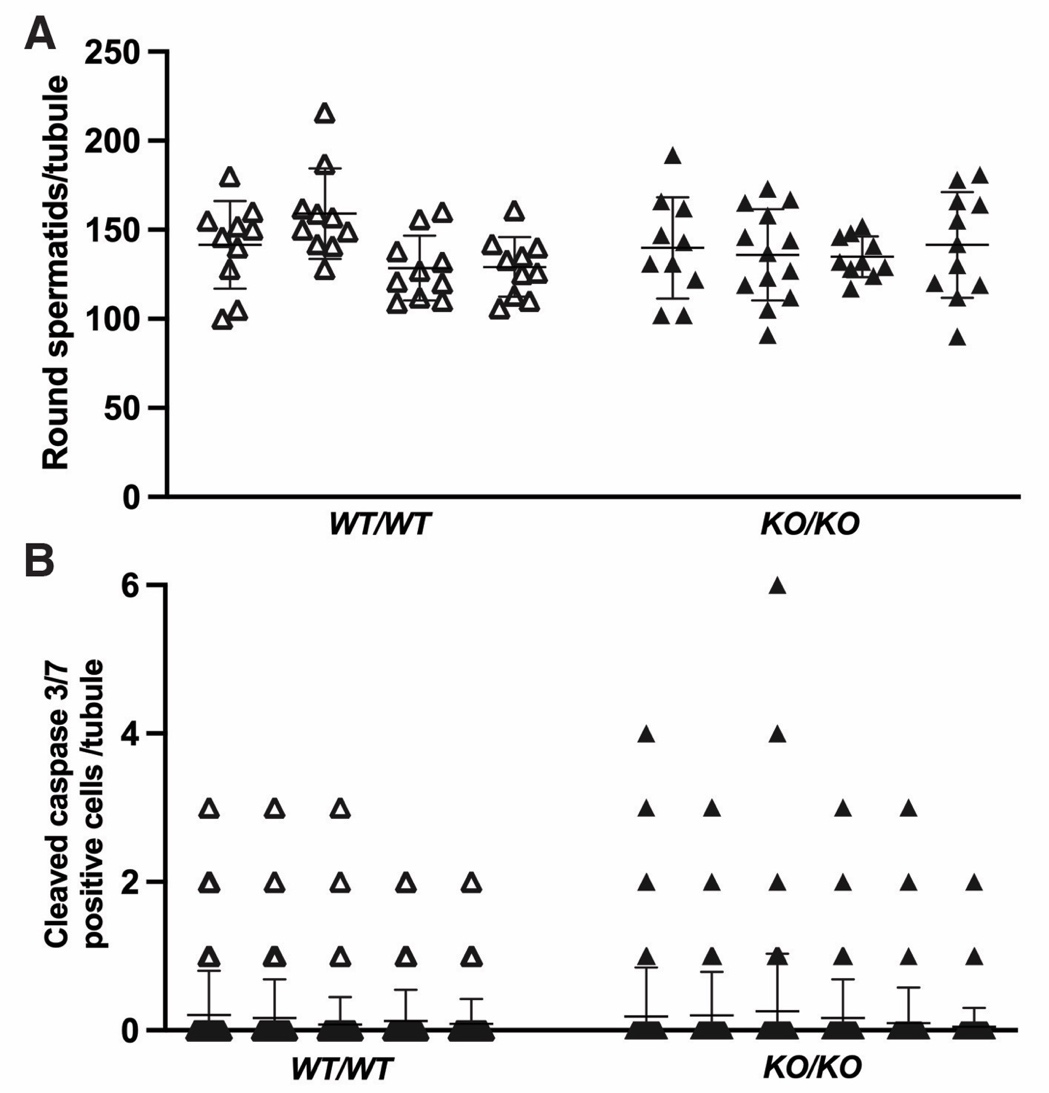


**Fig. S2. Loss of CCDC112 function does not significantly affect germ cell apoptosis or round spermatid number.**

(A) Round spermatid number per stage VIII tubule was assessed in *Ccdc112^WT/WT^* and *Ccdc112^KO/KO^* testes (n = 4/genotype). (B) Germ cell apoptosis in *Ccdc112^KO/KO^* testes was assessed via cleaved caspase 3 and 9 staining (n = 5–6/genotype). Lines denote mean ± SD.

**
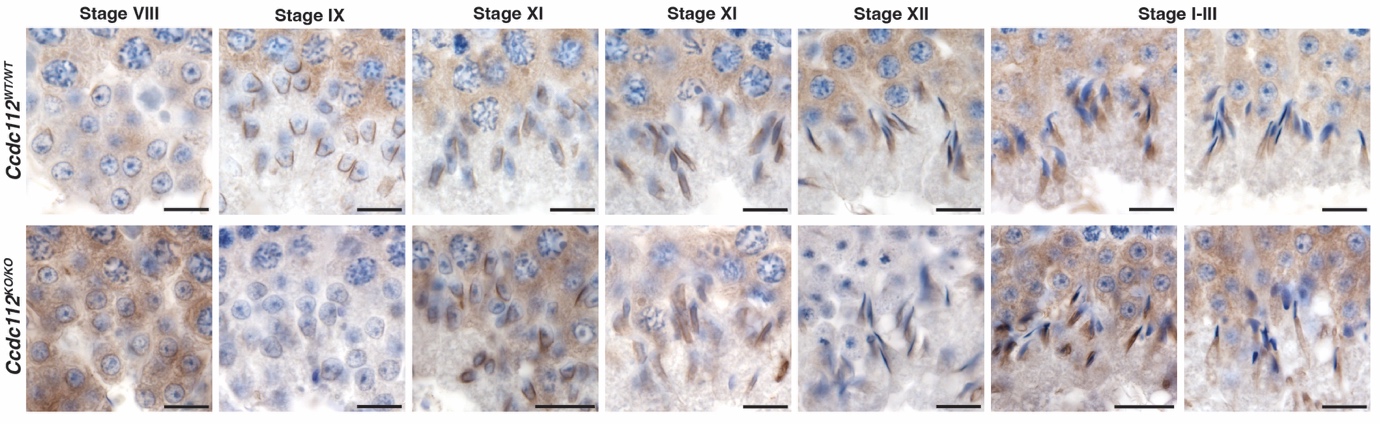
**

**Fig. S3. Manchette formation and movement does not require CCDC112**

Assessment of manchette formation via α-tubulin, a manchette microtubule marker, staining of *Ccdc112^WT/WT^* and *Ccdc112^KO/KO^* testis sections. Manchette assembly, elongation, descension, and disassembly appears comparable between genotypes. Scale bars = 20 µm.


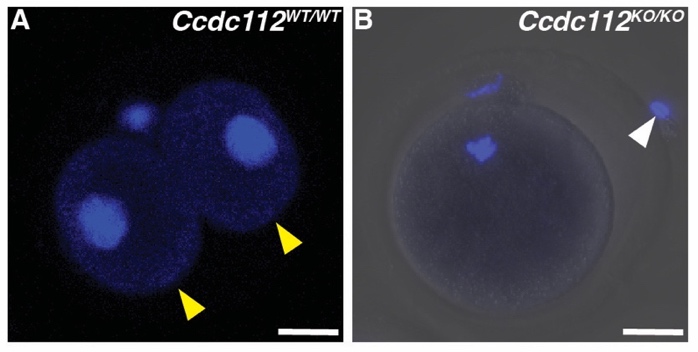


**Fig. S4. CCDC112 is essential for sperm penetration of the zonae pellucidae of oocytes *in vitro***

Immunofluorescence staining of oocytes 16 hours post insemination with *Ccdc112^WT/WT^* and *Ccdc112^KO/KO^* mouse sperm as marked by DAPI. Oocytes fertilized with *Ccdc112^WT/WT^* mouse sperm developed to a two-cell stage (A; yellow arrowheads). Conversely, *Ccdc112^KO/KO^* mouse sperm were mostly unable to penetrate the zonae pellucidae (B; white arrowhead). Scale bars = 20 µm.


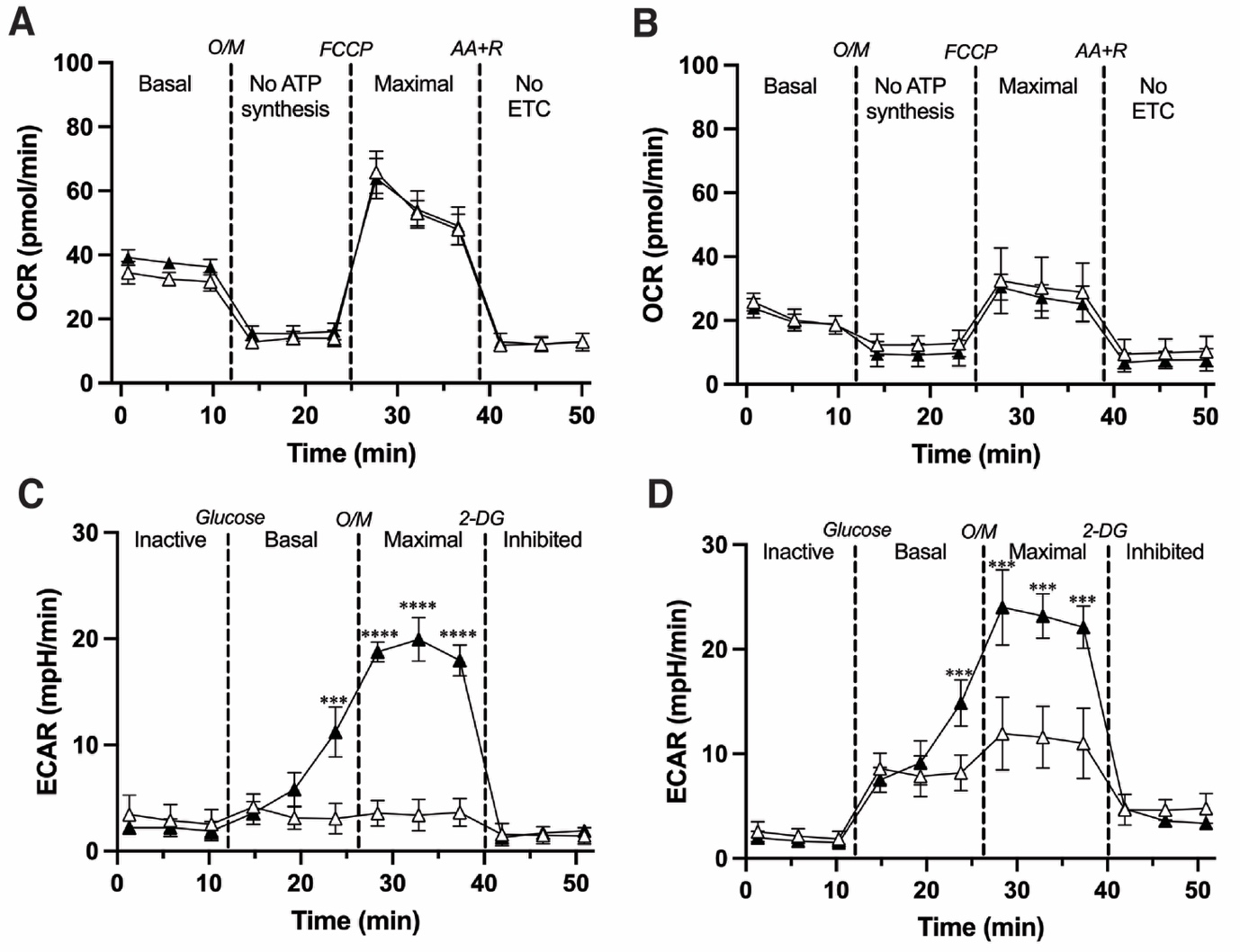


**Fig. S5. The effect of CCDC112 presence or absence in non-capacitated and capacitated sperm metabolism.**

Mitochondria stress test assay on *Ccdc112^WT/WT^* (A) and *Ccdc112^KO/KO^* (B) non-capacitated (white triangles) and capacitated mouse sperm (black triangles) (n = 3/genotype). Glycolytic flux Seahorse assay on *Ccdc112^WT/WT^* (C) and *Ccdc112^KO/KO^* (D) non-capacitated and capacitated mouse sperm samples (n = 3 mice/genotype). OCR = oxygen consumption rate; ECAR = extracellular acidification rate; O/M = oligomycin; AA + R = antimycin A and rotenone; 2-DG = 2-Deoxy-D-Glucose. Lines denote mean ± SD; *** P < 0.001, **** P < 0.0001.

**
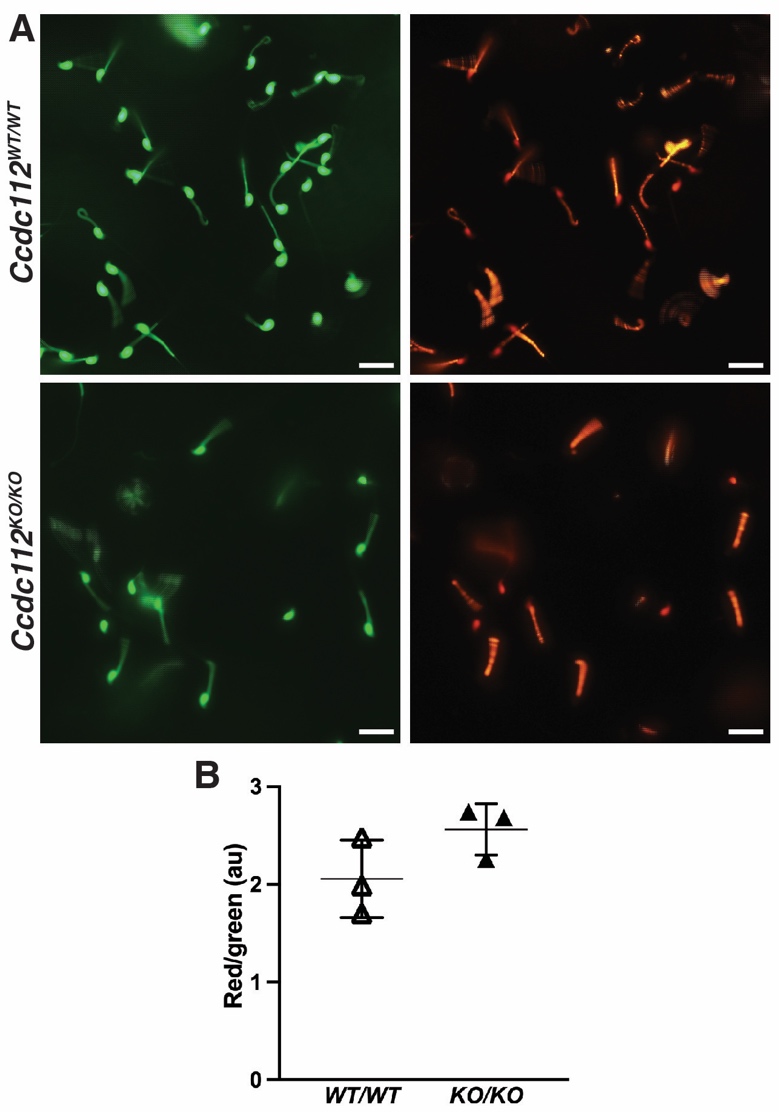
**

**Fig. S6. Loss of CCDC112 does not compromise mitochondria membrane potential.**

(A) Representative images of JC1 staining sperm from *Ccdc112^WT/WT^* and *Ccdc112^KO/KO^* mice. (B) Assessment of sperm mitochondrial potential via JC1 staining in *Ccdc112^WT/WT^* (white triangles) and *Ccdc112^KO/KO^* (black triangles) mice. Lines denote mean ± SD. Scale bars in A = 20 µm.

**Movies S1 and S2. CCDC112 is essential for a normal sperm flagella waveform**

Representative videos of *Ccdc112^WT/WT^* (1) and *Ccdc112^KO/KO^* (2) mouse sperm flagella waveform over 4.5 seconds.
